# Roles of RIN and ethylene in tomato fruit ripening and ripening‐associated traits

**DOI:** 10.1111/nph.16362

**Published:** 2019-12-31

**Authors:** Shan Li, Benzhong Zhu, Julien Pirrello, Changjie Xu, Bo Zhang, Mondher Bouzayen, Kunsong Chen, Donald Grierson

**Affiliations:** ^1^ College of Agriculture & Biotechnology Zhejiang University Zijingang Campus Hangzhou 310058 China; ^2^ Zhejiang Provincial Key Laboratory of Horticultural Plant Integrative Biology Zhejiang University Zijingang Campus Hangzhou 310058 China; ^3^ College of Food Science and Nutritional Engineering China Agricultural University Beijing 100083 China; ^4^ GBF Laboratory University of Toulouse INRA Castanet‐Tolosan 31320 France; ^5^ Plant and Crop Sciences Division School of Biosciences University of Nottingham Sutton Bonington Campus Loughborough LE12 5RD UK

**Keywords:** carotenoid, cell wall, MADS‐RIN, post‐harvest softening, ripening initiation, *Solanum lycopersicum* (tomato), system‐2 ethylene (ET), volatile

## Abstract

- RIPENING INHIBITOR (RIN)‐deficient fruits generated by CRISPR/Cas9 initiated partial ripening at a similar time to wild‐type (WT) fruits but only 10% WT concentrations of carotenoids and ethylene (ET) were synthesized. RIN‐deficient fruit never ripened completely, even when supplied with exogenous ET. The low amount of endogenous ET that they did produce was sufficient to enable ripening initiation and this could be suppressed by the ET perception inhibitor 1‐MCP.
- The reduced ET production by RIN‐deficient tomatoes was due to an inability to induce autocatalytic system‐2 ET synthesis, a characteristic feature of climacteric ripening. Production of volatiles and transcripts of key volatile biosynthetic genes also were greatly reduced in the absence of RIN.
- By contrast, the initial extent and rates of softening in the absence of RIN were similar to WT fruits, although detailed analysis showed that the expression of some cell wall‐modifying enzymes was delayed and others increased in the absence of RIN.
- These results support a model where RIN and ET, via ERFs, are required for full expression of ripening genes. Ethylene initiates ripening of mature green fruit, upregulates *RIN* expression and other changes, including system‐2 ET production. RIN, ET and other factors are required for completion of the full fruit‐ripening programme.

## Introduction

Fleshy fruits evolved both to protect developing seeds and aid seed dispersal. They have traditionally been classified into climacteric (e.g. apples, pears, bananas, melons and tomato) and nonclimacteric (e.g. pineapple, strawberry, citrus) types. Climacteric fruits such as tomato show a characteristic rise in respiration, the respiratory climacteric, and a marked rise in ethylene (ET) production at the onset of ripening. The tomato (*Solanum lycopersicum*) has been a model for understanding the molecular basis of ripening for more than 40 yr because of its commercial importance but also because it is well characterized genetically, several crops can be grown year‐round in controlled environments, it can be relatively easily transformed, and several important natural mutations have been identified that affect different aspect of ripening (Giovannoni, 2007; Klee and Giovannoni, 2011). During fruit growth, the cells enlarge and accumulate reserves and at maturity multiple changes are initiated in a coordinated manner which alter colour, flavour, texture and aroma. This ripening process converts a hard, unappealing fruit into a colourful nutritious product that attracts birds, animals and humans to aid in seed dispersal (Giovannoni, 2004).

The transcription factor MADS‐RIN (RIN, RIPENING INHIBITOR) has long been viewed as a master regulator of ripening. RIN affects accumulation of many gene transcripts (Giovannoni, 2004), proteins and their post‐translational degradation (Qin *et al.*, 2012; Wang *et al.*, 2014). Recent advances in knowledge concerning the transcription factors that regulate climacteric fruits ripening (Ito *et al.*, 2017; Gao *et al.*, 2018; Li *et al.*, 2018; Wang *et al.*, 2019), including discrepancies between the phenotypes of natural mutants and CRISPR/Cas9‐induced mutations of *ripening inhibitor* (*rin)*, *non‐ripening (nor)* and *colourless non‐ripening (Cnr)*, have called into question the precise roles of these regulatory genes in the ripening control network (Ito *et al.*, 2017; Gao *et al.*, 2018; Li *et al.*, 2019; Wang *et al.*, 2019). The *rin* mutation was originally thought to correspond to a loss‐of‐function event (Vrebalov *et al.*, 2002) but is now known to generate an active hybrid transcription factor, RIN‐MC, which has a repressor activity (Ito *et al.*, 2017; Li *et al.*, 2018). RIN is still critical for progression of ripening but is not required for ripening initiation (Ito *et al.*, 2017; Li *et al.*, 2018), and the factor actually responsible for ripening initiation in the absence of RIN has not been identified.

Ethylene has long been implicated in ripening control. All plants produce some ET, but this can increase 100‐fold as climacteric fruit transition from low concentration (system‐1) ET, which is self‐inhibitory, to a major burst of autocatalytic ET synthesis, called system‐2 ET (McMurchie *et al.*, 1972; Grierson, 2013, 2014) during ripening. Nonclimacteric fruits also respire and evolve low concentrations of ET, but they do not show a climacteric burst. Analysis of natural mutants which have pleiotropic effects on multiple genes has identified ripening regulators such as MADS‐RIN, NAC‐NOR and the squamosa‐box binding protein CNR, some of which act at least partly independently of ET (Martel *et al.*, 2011; Kumar *et al.*, 2012; Giovannoni *et al.*, 2017). Activator‐ and repressor‐ET response factors (ERFs) and auxin response factors (ARFs) operate downstream of their respective hormone signalling pathways to regulate gene expression and hormone cross‐talk (Liu *et al.*, 2015, 2018). Ethylene promotes ripening (Tucker *et al.*, 2017), abscisic acid also may play a part (Chen *et al.*, 2016; Mou *et al.*, 2016), whereas auxin tends to inhibit ripening (Shin *et al.*, 2019). The way in which this complex network of transcription factors and hormones operates to control the expression of many ripening genes is still being actively investigated (Li *et al.*, 2019), but it is known that together they control production of enzymes for biosynthesis of coloured pigments such as carotenoids in the chromoplasts (or anthocyanins in the vacuoles in other fruits), multiple cell wall softening enzymes, metabolism of acids and sugars affecting taste, and production of multiple aroma volatiles (Giovannoni, 2004; Grierson, 2013; Klee and Tieman, 2018; Li *et al.*, 2019).

Recent work describing the removal of RIN by CRISPR/Cas9 or RNAi strategies (Ito *et al.*, 2017; Li *et al.*, 2018) has shown that the precise role of RIN in climacteric ripening needs further clarification. Here we characterize new RIN‐deficient *Ailsa Craig* tomato fruits obtained by CRISPR/Cas9 technology and show that they produce sufficient endogenous ET to induce ripening and this RIN‐independent initiation of ripening is inhibited by the ET perception inhibitor 1‐methylcyclopropene (1‐MCP). Ethylene production is low because RIN‐deficient fruits are unable to induce autocatalytic system‐2 ET production and they also are deficient in volatiles and carotenoids and transcripts associated with these pathways. Strikingly, extensive softening occurs independently of RIN, which contrasts strongly with the original *rin* mutant phenotype. Moreover, late softening of RIN‐deficient fruits coincides with the delayed accumulation of several cell wall enzymes including *MAN1*, *Mside7*, *MAN4a, TBG4, PG* and *PME2.1*. These results support a model in which ET is required for the initiation of ripening and the combined action of RIN and ET is required for the progression and completion of different facets of the ripening process.

## Materials and Methods

### Plasmid construction

The target site for CRISPR/Cas9‐mediated RIPENING INHIBITOR (RIN) mutagenesis was selected using the CRISPR‐P program (http://cbi.hzau.edu.cn/cgi-bin/CRISPR) (Supporting Information, Fig. S1a). The 20‐bp oligos were cloned into AtU3d and AtU3b vectors and the sgRNA expression cassettes assembled into pYLCRISPR/Cas9‐Ubi‐H binary plasmid by Golden Gate ligation (Ma *et al.*, 2015). *Agrobacterium tumefaciens*‐mediated transfer of T‐DNA was used for stable transformation of tomato (Sun *et al.*, 2006; Kimura and Sinha, 2008). For the mutation analysis, genomic DNA was extracted from young tomato leaves using a Plant Genomic DNA Kit (Tiangen, China) and used as a template to amplify the *RIN* fragment using PCR and the fragments sent for sequencing. The primer pairs used for vector construction and mutation analyses are listed in Table S1.

### Plant material and growth conditions

Wild‐type (WT) tomato (*Solanum lycopersicum Alisa Craig*, AC) and RIN‐CRISPR seedlings were grown in a glasshouse under long‐day conditions (16 h : 8 h, light : dark photperiod) at a temperature of 26°C. For gene expression analysis, organs were collected, frozen in liquid N_2_, and stored at −80°C until RNA extraction. Three independent samplings were performed for each measurement.

### Tomato fruit nuclei isolation and Western blotting

Nuclei were isolated from tomato fruits picked at B + 5 stage and assayed for RIN protein. Fruit samples were ground into a powder under liquid N_2 _and the mixture was extracted with buffer (0.25 M sucrose, 10 mM Tris‐HCl pH7.5, 1 mM MgCl_2_, 0.5% PVP, 0.5% Triton X‐100, Roche protease inhibitor tablet) and the suspension filtered using miracloth (475855; Millipore, Pittsburgh, PA, USA). After centrifugation at 10 000 ***g*** for 10 min, the precipitate was washed with extraction buffer and centrifuged again at 10 000 ***g*** for 10 min, and the pellet was resuspended in percoll buffer (0.25 M sucrose, 95% Percoll, 10 mM Tris‐HCl pH7.5, Roche protease inhibitor tablet). The floating layer was collected after centrifugation at 10 000 ***g*** for 10 min, diluted to 30% with extraction buffer, centrifuged at 10 000 *g* for 10 min, to pellet the nuclei and stored at −80°C or used for SDS‐PAGE assay.

Western blotting was carried out as described (Li *et al.*, 2018). Briefly, protein extracts were separated on 10% SDS‐PAGE gels and transferred to a polyvinylidene fluoride (PVDF) membrane blocked in 5% nonfat milk for 2 h at room temperature. A specific polyclonal antibody produced in rabbit raised against the C‐terminal end of RIN (amino acids 75–242) was added in a ratio of 1 : 1000 and incubated for 2 h at room temperature. Membranes were washed with Tris‐buffered saline plus Tween‐20 three times, 15 min each time. The anti‐rabbit horseradish peroxidase secondary antibody was added at a ratio of 1 : 10 000 and incubated for 2 h at room temperature. After three washes with Tris‐buffered saline plus Tween‐20, the membranes were visualized using a horseradish peroxidase‐enhanced chemiluminescence system.

### Ethylene production measurement

For the measurement of ethylene (ET) production, each fruit was placed in a sealed gas‐tight 300 ml container at 25°C for 1 h, and a 1 ml headspace gas sample was analyzed using GC (6890N GC system; Agilent, Folsom, CA, USA) equipped with a flame ionization detector (Ma *et al.*, 2016).

### Colour measurement

A Hunter Lab Mini Scan XE Plus colorimeter (Hunter Associates Laboratory Inc., Reston, VA, USA) with the CIE L*a*b colour system was chosen for pericarp colour assay (Komatsu *et al.*, 2016). At least six biological replicates were used for each assay.

### Carotenoid content assay

Carotenoid extraction followed the methods reported by Xu *et al.* (2006); 100 mg tomato fruit samples were ground to a powder and frozen at −80°C, 250 μl methanol was added, vortexed to mix, followed by 500 μl chloroform, vortexed again and 250 μl 50 mM Tris buffer (pH 7.5, containing 1 M NaCl) was added, followed by vortexing. After centrifugation (15 000 ***g*** for 10 min at 4°C), the lower chloroform phase was collected. The chloroform extraction was repeated two or three times and the chloroform phases combined and dried under flowing N_2_. The residue was dissolved in 100 μl ethyl acetate (HPLC grade), and 50 μl transferred to HPLC sample analysis tubes. Carotenoid content was assayed according to the methods reported by Zheng *et al.* (2015): A volume of 20 μl for each sample was absorbed for HPLC analysis, carried out using a Waters liquid chromatography system (e2695) equipped with a photodiode array (PDA) detector (2998). A C30 carotenoid column (250 mm × 4.6 mm; YMC, Japan) was used to elute the carotenoids with a methanol: H_2_O (9 : 1, v/v, eluent A) solution and methyl tert‐butyl ether (MTBE) (100%, eluent B) solution containing 0.01% (w/v) butylated hydroxytoluene (BHT). The linear gradient program was performed as follows: 8% B to 25% B for 30 min, 25% B to 70% B for 5 min, 70% B for 5 min, and back to the initial 8% B for re‐equilibration for 10 min. The flow rate was 1 ml min^−1^. To avoid light degradation of carotenoids the extraction and analysis were performed under subdued light.

### Firmness measurement

The firmness of the pericarp was assayed using a penetrometer (TA‐XT2i texture analyzer; Stable Micro Systems, Stable Micro Systems Ltd, Surrey, UK) according to the manufacturer's instructions. At least six biological replicates were used for each assay.

### Volatiles assays

Measurements of volatiles were carried out according to Zhang *et al.* (2010), with modifications. First, 5 g of frozen flesh tissue was ground in liquid N_2 _and transferred to a 15‐ml vial containing 5 ml of saturated sodium chloride solution. Before vials were sealed, 20 μl of 2‐octanol (0.8 mg ml^−1^) was added as an internal standard and vortexed for 10 s.

For solid‐phase microextraction (SPME), samples then were equilibrated at 40°C for 30 min before being exposed to a fiber coated with 50/30 μm DVB/CAR/PDMS (Supelco Co., Bellefonte, PA, USA). Volatiles were subsequently desorbed over 5 min at 230°C into the splitless injection port of the GC‐flame ionization detector (FID). An Agilent 7890A GC equipped with an FID and a DB‐WAX column (30 m × 0.32 mm, 0.25 μm internal diameter; J&W Scientific, Folsom, CA, USA) was used for volatile analysis. Chromatography conditions were as follows: injector, 230°C; initial oven temperature, 34°C held for 2 min, increased by 2°C min^−1^ to 60°C, then increased by 5°C min^−1^ to 220°C, and held for 2 min. Nitrogen was used as carrier gas at 1.0 ml min^−1^. Volatiles were identified by comparison with retention times of authentic standards. Further identification of volatile compounds was by capillary gas chromatography‐mass spectrometry (GC‐MS) (7890A‐5975C) performed using an HP‐5 MS column (30 m × 0.25 mm, 0.25 μm; J&W Scientific, Folsom, CA). Injection port temperature was 240°C, with a split ratio of 5 : 1. Helium was used as the carrier gas at a rate of 1.0 ml min^−1^. The column temperature was held at 40°C for 2 min, increased by 5°C min^−1^ to 60°C, then increased by 10°C min^−1^ to 250°C, and held for 5 min. MS conditions were as follows: ion source, 230°C; electron energy, 70 eV; multiplier voltage, 1247 V; GC‐MS interface zone, 280°C; and a scan range, 30–250 mass units. Volatiles were identified on the basis of a comparison of their electron ionization (EI) mass spectra to published data and data from authentic standards. Quantitative determination of compounds was carried out using the peak of the internal standard as a reference value and calculated on the basis of standard curves constructed with authentic compounds.

### Ethylene, 1‐methylcyclopropene (1‐MCP) and propylene treatment

Tomato fruits at the mature green (MG) stage, before any sign of colour change, were placed in an air‐tight 1‐l plastic container with 100 ppm ET, 1000 ppm propylene or 10 ppm 1‐MCP. 1000 ppm propylene is equivalent to 10 ppm ET treatment (McMurchie *et al.*, 1972) and is used in order to distinguish it from endogenous ET production by GC equipment. The treatment was conducted continually in an incubator under a 16 h : 8 h, light : dark photoperiod at 25°C, with at least three biological replicates for each treatment. RIN‐CRISPR tomato fruits treated with ET for 48 h, and control WT and RIN‐CRISPR treated with air, were chosen for gene expression assay using qRT‐PCR. The gas environments (air, ET, propylene, 1‐MCP) were replenished every 24 h.

### RNA isolation and quantitative reverse transcription (qRT)‐PCR

Isolation of RNA from tomato fruit pericarp at different ripening stages was as described previously (Zhu *et al.*, 2015). Total RNA extraction from tomato fruit pericarp was carried out using Trizol reagent, and RNA integrity was verified by 1.5% (v/v) agar gel electrophoresis. Genomic DNA was removed from RNA preparations by digestion with DNase I (Invitrogen, cat. no. AM1907), and RNA quality and quantity were confirmed by spectrophotometry (Thermo Scientific, Waltham, MA, USA; NanoDrop 1000). RNA was reverse‐transcribed into cDNA using cDNA synthesis kit (Bio‐RAD, cat. no. 1708890) according to the manufacturer's instructions. qRT‐PCR was conducted using FastStart Essential DNA Green Master (Roche, cat. no. 06402712001) with a LightCycler480 (Roche). Relative gene expression values were calculated using the 2^‐ΔΔCt^ method (Livak and Schmittgen, 2001). The tomato *ACTIN* gene (Solyc03g078400) was used as an internal reference gene. At least three biological replicates were included for each point, and each replicate was from independent sampling. The primer pairs used in qRT‐PCR analyses are listed in Table S2.

### Water loss

The water lost by tomato fruits was calculated as FW (%) = fruit weight (g) – fresh fruit weight (g)/ fresh fruit weight (g) × 100%. More than ten biological replicates were used for each assay.

### Promoter sequence and motif assay

Promoter sequences 2.0 kb in length were downloaded from Sol Genomics Network (https://solgenomics.net/), various CArG‐box elements were from Fujisawa *et al.* (2013). The GCC‐box, a characteristic *cis*‐element binding site for ERFs, was from Licausi *et al.* (2013). An AP2/ERF binding motif, ATCTA was from Welsch *et al.* (2007).

### Statistical analysis

Microsoft excel 2010 and Spss (IBM SPSS Statistics, v.22; SPSS Inc., Chicago, IL, USA) were used for statistical analyses. Duncan's multiple range test was used (*P* < 0.05).

## Results

### Generation of RIN mutants using the CRISPR/Cas9 gene‐editing system

Recent studies using CRISPR/Cas9 or RNAi have shown that ripening initiation is not completely inhibited in RIN‐deficient tomato fruit (Ito *et al.*, 2017; Li *et al.*, 2018), however, the possible role of ET in ripening initiation in the absence of RIN has not been investigated. To clarify the precise role of RIN and other factors controlling the different aspects of climacteric ripening, we generated new RIN loss‐of‐function tomato lines, using the CRISPR/Cas9 gene editing system. Two genomic sites were targeted for cleavage (Fig. S1a), and multiple independent transgenic plants were genotyped by the direct sequencing of PCR products from genomic DNA flanking the target sites. After two generations of screening, ten lines were identified carrying homozygous mutations in exon 2: deletion of 432 bp corresponding to the entire exon 2 (RIN‐CRISPR‐1); deletion of 11 bp (RIN‐CRISPR‐2); insertion of 1 bp (RIN‐CRISPR‐3); deletion of 1 bp (RIN‐CRISPR‐4); deletion of 2 bp (RIN‐CRISPR‐5); insertion of 1bp (RIN‐CRISPR‐6); insertion of 1 bp (RIN‐CRISPR‐7, RIN‐CRISPR‐8); deletion of 2 bp (RIN‐CRISPR‐9); and changes in 4 bp (RIN‐CRISPR‐10) (Fig. S1b). Hereafter, these 10 are referred to as RIN‐deficient lines. The manipulations introduced premature stop codons in the *RIN* coding sequence, which led to predicted truncated RIN polypeptides of 68, 74, 78, 94, 77, 97, 78, 78, 96 and 242 (with 21 changes) amino acids of the MADS domain (Fig. S1b), compared to the 242 amino acids of WT RIN native protein.

**Figure 1:**
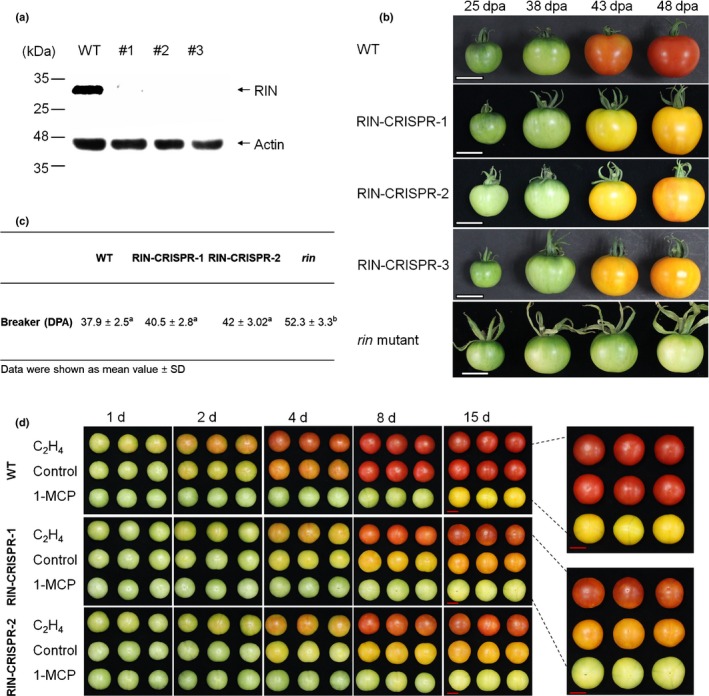
Phenotypes, RIPENING INHIBITOR (RIN) protein content and ethylene (ET) response of wild‐type (WT) and RIN‐deficient tomato (*Solanum lycopersicum*) fruits. (a) The detection of RIN protein by Western blotting in WT but not RIN‐CRISPR fruits, using RIN‐poly‐antibody. Actin was used as internal control. (b) Tomato fruits from the RIN‐MC mutant (*rin* mutant) and three RIN‐CRISPR lines (RIN‐CRISPR‐1‐3) generated by CRISPR/Cas9‐mediated mutagenesis of the *RIN* gene are shown at 25 d after anthesis (dpa), 38 dpa, 43 dpa and 48 dpa, and compared to WT fruit. Bar, 2 cm. (c) Number of days from anthesis to ripening of *S.* *lycopersicum Alisa Craig *(AC), RIN‐CRISPR and *rin* mutant tomato fruits. (d) Effect of exogenous ET and ET perception inhibitor 1‐MCP treatment on ripening progression of RIN‐CRISPR tomato fruit. WT and RIN‐CRISPR tomato fruits were picked at MG stages and treated and. replenished daily with ET (100 ppm) and 1‐MCP (10 ppm) or air continually for up to 15 d. Fruits in horizontal rows are biological replicates. Enlarged photos of representative samples are shown compared to WT fruits on the right. Bar, 2 cm.

In order to assess the presence of RIN protein in the edited tomato lines, we used antibodies specific to the C‐terminal region (sites 158–242 AAs), which encodes the activation domain of the RIN protein (Qin *et al.*, 2012; Martel *et al.*, 2011; Ito *et al.*, 2008) as the N‐terminal MADS‐box domain is highly conserved among MADS‐box proteins. Western blot analysis revealed that full‐length RIN protein could only be detected in WT and was absent from RIN‐CRISPR fruits (Fig. 1a). All of the lines bearing premature stop codons in the *RIN* gene displayed similar altered ripening phenotypes. Three of these were studied more closely and two representative lines (named RIN‐CRISPR‐1, ‐2) used for detailed physiological analyses and molecular studies (Fig. S2).

### RIN‐deficient fruit reveal that ripening can be initiated independently from RIN

Assessing the ripening behaviour of RIN‐deficient fruits revealed that all three independent mutant lines (RIN‐CRISPR‐1, ‐2, ‐3) initiated ripening at approximately the same time as WT fruits and there was no obvious delay in ripening onset (Fig. 1b,c). This contrasted with the situation for *rin* mutant fruits, where initiation of ripening was delayed by 14 d or more (Fig. 1c) and fruits only ever turned a pale yellow colour and did not soften. The RIN‐deficient fruits not only ripened earlier than *rin* mutant fruit but became a deep yellow/orange colour and softened, the *rin* mutant fruit slowly became pale yellow but did not soften, whereas WT fruits softened and turned red (Fig. 1b).

When they were allowed to ripen for longer, RIN‐CRISPR fruits became orange. This appeared to vary slightly in different fruits, according to age and position on the plant, suggesting that they might be sensitive to some other factor(s). The ability of RIN‐deficient fruit to undergo ripening in response to ET was investigated. Detached mature green (MG) WT and RIN‐CRISPR fruits were treated with either external ET (100 ppm) or its competitive inhibitor 1‐methylcyclopropene (1‐MCP) (10 ppm). External ET accelerated red colour development in WT fruit with a visible difference after 2 d treatment, whereas 1‐MCP inhibited this process very significantly for ≥ 15 d (Fig. 1d). Ethylene also accelerated colour change in RIN‐CRISPR fruits, which became faintly orange‐red, whereas 1‐MCP significantly inhibited the transition from green to yellow for many days (Fig. 1d). Although supplying ET externally to picked fruit induced slight red coloration in RIN‐deficient fruits, the response was weaker than in WT fruits and occurred more slowly (Fig. 1d). These results indicated that RIN‐CRISPR fruits are able to respond to ET by enhancing pigment accumulation during ripening, but without approaching the concentrations displayed by WT fruits.

### RIN‐CRISPR fruits are deficient in climacteric ET production and unable to induce system‐2 ET synthesis

**Figure 2:**
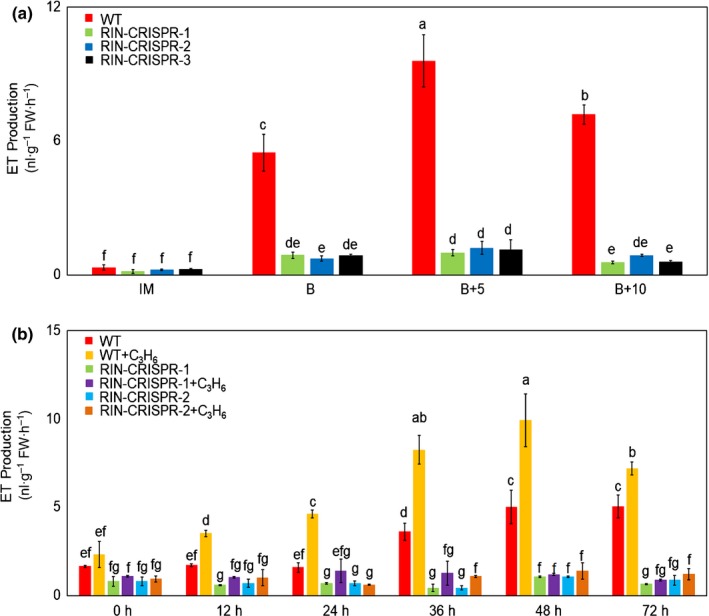
Ethylene (ET) production and response of wild‐type (WT) and RIPENING INHIBITOR (RIN)‐deficient tomato fruits to propylene. (a) Ethylene production in WT and RIN‐CRISPR tomato fruits at immature (IM), breaker (B), breaker + 5 (B + 5) and breaker + 10 (B + 10) stages, measured by GC (average of three biological replicates). (b) Ethylene production of tomato WT and RIN‐CRISPR fruits treated with propylene. Fruit were picked at MG stages and treated with propylene (1000 ppm) (equivalent to 10 ppm ET treatment) (McMurchie *et al*., 1972) or air (control) continually for 12, 24, 36, 48 and 72 h to test for induction of endogenous system‐2. Propylene was replenished every 12 h. The error bars represent mean ± SD, the lowercase letters indicate significant difference at *P* = 0.05.

In order to address whether the reduced ET production of RIN‐deficient tomatoes is due to their inability to induce autocatalytic system‐2 ET synthesis, a characteristic feature of normal climacteric fruit ripening, we treated RIN‐deficient fruit with propylene, an analogue which mimics the hormone effect of ET. In the absence of treatment, WT fruits showed a characteristic burst of ET synthesis after the onset of ripening, whereas ET production by RIN‐CRISPR fruits was substantially inhibited compared to WT (Fig. 2a). Thereafter, detached WT and RIN‐CRISPR MG fruits were challenged by continuous treatment with 1000 ppm propylene which is equivalent to 10 ppm ET treatment (McMurchie *et al.*, 1972). An obvious burst of ET production was detected after 24 h with WT fruits, which reached a peak at 48 h (Fig. 2b). No increase in ET production was detected, however, in RIN‐CRISPR fruits even after 72 h treatment (Fig. 2b), indicating that the autocatalytic system‐2 ET burst could not be induced by propylene treatment in the absence of RIN.

Several differences between RIN‐CRISPR and WT fruits were found in the expression of genes related to ET biosynthesis, perception and signalling (Fig. S3). Transcripts of *ACC synthase* (*ACS*) and *ACC oxidase* (*ACO*), the ET biosynthesis genes, all were greatly reduced in RIN‐deficient fruits, compared to WT fruits. *ACS2* and *ACS4* transcripts, the main *ACS* genes that increase during ripening, accumulated to < 20% of their concentrations in WT respectively, whereas *ACO1* and *ETR3/NR* transcripts reached about 40% and 50% of WT concentrations, respectively. This is consistent with the suggestion that there is a dual control of these genes (Yokotani *et al.*, 2009) and it is noteworthy that they are known to be specifically involved in system‐2 ET synthesis. Moreover, the *ACS* genes affected in RIN‐deficient fruits are those identified previously among the RIN targets (Fujisawa *et al.*, 2012; Zhong *et al.*, 2013). The classic ET‐induced tomato genes *E4* and *E8* transcripts also were greatly reduced (Fig. S3; Lincoln and Fischer, 1988).

### Pigment accumulation is impaired in RIN‐deficient fruit

**Figure 3:**
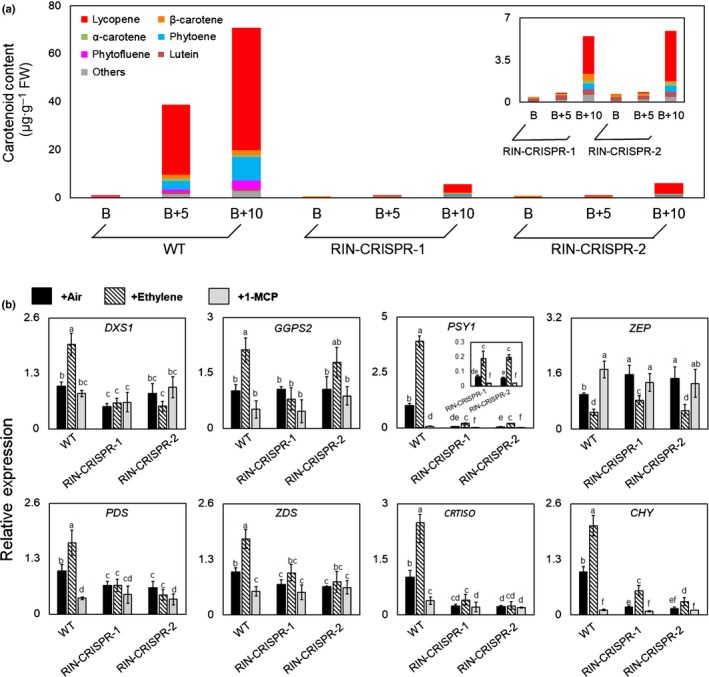
Comparison of changes in pigment production and quantitative reverse transcription (qRT)‐PCR assay of genes involved in pigment accumulation in wild‐type (WT) and RIPENING INHIBITOR (RIN)‐deficient tomato fruits. (a) Carotenoid content of WT and RIN‐CRISPR measured by HPLC (average of three biological replicates). (b) qRT‐PCR of gene transcripts involved in carotenoids biosynthesis (average of three biological replicates). Relative transcript levels are expressed relative to the tomato *ACTIN* gene internal control, expressed as 2^‐ΔΔCt^ (Livak and Schmittgen, 2001). *1‐d‐deoxyxylulose 5‐phosphate synthase* (*DXS1*); *geranylgeranyl pyrophosphate synthases* (*GGPS2*); *phytoene synthases 1* (*PSY1*); *phytoene desaturase* (*PDS*); *zeta‐carotene desaturase* (*ZDS*); *carotene isomerase* (*CRTISO*); *non‐heme hydroxylases* (*SlBCH2/CHY*); *zeaxanthin epoxidase* (*ZEP*). The error bars represent mean ± SD, the lowercase letters indicate significant difference at *P* = 0.05. Genes identified as having low level of transcripts by RNA‐Seq (Li *et al.*, 2018) were not measured.

Pigment accumulation is a major fruit ripening trait and colour change depends on the degradation of chlorophyll, and the synthesis and accumulation of the coloured carotenoids lycopene and β‐carotene. The tomato fruits pericarp colours were measured with a colorimeter using the CIE L*a*b colour system (Komatsu *et al.*, 2016). The a* value refers to the degree of red to green, determined by the degradation of chlorophyll and the accumulation of carotenoids, such as β‐carotene and lycopene, which produce the characteristic yellow, orange and red coloration (Luo *et al.*, 2013; Fig. S4). At B + 5 and B + 10 stages WT fruits turned red gradually. By contrast, the three RIN‐CRISPR mutants turned yellow and slowly developed an orange tinge 1–2 wk after ripening onset (Fig. 1). The carotenoid content and constituents were determined quantitatively by HPLC in WT and RIN‐CRISPR fruits at B, B + 5 and B + 10 stages. WT fruits accumulated more than a 10‐fold higher concentration of lycopene, the main red colour than RIN‐CRISPR fruits, which, in addition to having low lycopene, accumulated no phytofluene, and very low amounts of phytoene and alpha‐carotene (Fig. 3a). This indicated how severely carotenoid accumulation is inhibited in RIN‐CRISPR fruits. By contrast, the *rin* mutant fruit remained yellow until B + 10 stage, with almost no detectable lycopene (Li *et al.*, 2018). Transcripts for the key enzymes *phytoene synthase1* (*PSY1*) (Bird *et al.*, 1991; Fray and Grierson, 1993) and *non‐heme hydroxylases* (*CHY/SlBCH2*) and *carotene isomerase* (*CRTISO*) were inhibited by 90%, 80% and 75%, respectively, in RIN‐deficient fruits and other transcripts of genes in the pathway (*1‐d‐deoxyxylulose 5‐phosphate synthase* (*DXS1*), *phytoene desaturase* (*PDS*), *zeta‐carotene desaturase* (*ZDS*)) were significantly reduced, whereas *geranylgeranyl pyrophosphate synthases* (*GGPS2*) was not affected and *zeaxanthin epoxidase* (*ZEP*) transcripts actually accumulated to a higher level in RIN‐deficient fruit. In agreement with the positive response to ET of RIN‐deficient fruits, transcripts of both *PSY1* (3.0‐ and 3.8*‐*fold increase in RIN‐CRISPR‐1 and RIN‐CRISPR‐2, respectively) and *CHY* (3.0‐ and 2.0‐fold increase in RIN‐CRISPR‐1 and RIN‐CRISPR‐2, respectively) accumulated to higher levels in response to ET treatment (Fig. 3b). These data indicate that colour change, a major ripening‐associated trait in tomato fruit, can be induced by ET in a RIN‐independent manner but high‐level accumulation of carotenoids requires RIN.

### Volatiles accumulation is impaired in RIN‐deficient fruit

**Figure 4:**
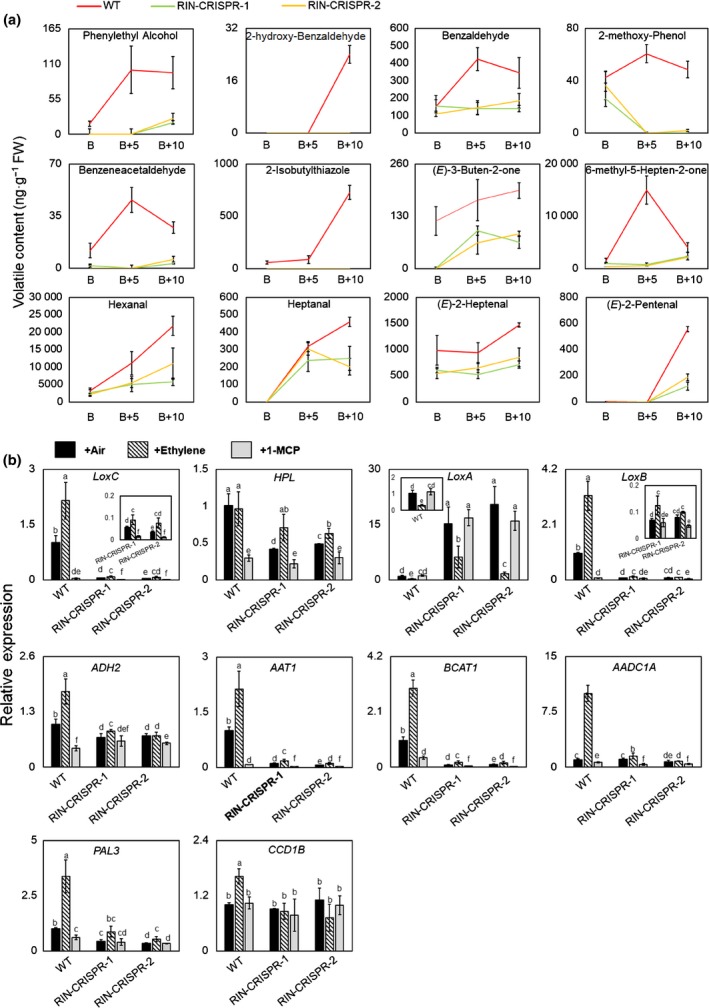
Volatile content and quantitative reverse transcription (qRT)‐PCR assay of genes involved in volatile formation during ripening of wild‐type (WT) and RIPENING INHIBITOR (RIN)‐deficient tomato fruits. (a) Aroma volatiles produced from different pathways including amino acid (phenylethyl alcohol, 2‐hydroxy‐benzaldehyde, 2‐methoxy‐phenol, benzeneacetaldehyde, benzaldehyde, 2‐isobutylthiazole), carotenoid (6‐methyl‐5‐hepten‐2‐one, (*E*)‐3‐buten‐2‐one), lipid (hexanal, heptanal, (*E*)‐2‐heptenal, (*E*)‐2‐pentenal). Volatiles were measured by GC or GC‐MS. (b) qRT‐PCR of gene transcripts involved in major tomato fruit pathways forming volatiles, relative to the expression of the tomato *ACTIN* gene as internal control, expressed as 2^‐ΔΔCt^ (Livak and Schmittgen, 2001). *Lipoxygenase* (*LoxA, LoxB, LoxC*); *hydroperoxide lyase* (*HPL*); *alcohol dehydrogenase 2* (*ADH2*); *alcohol acetyltransferase 1* (*AAT1*); *branched‐chain aminotransferases (BCAT1)*; *l‐phenylalanine ammonia lyase* (*PAL3*); *aromatic amino acid decarboxylase (AADC1A); carotenoid cleavage dioxygenase 1 (CCD1B).* RNA‐Seq has shown previously that genes such as carboxymethylesterase (carboxylesterase 1 (*CXE1*)); *PAL2,4,6* have only low transcript levels and *PAL1,5* are unaffected if *RIN* is silenced (Li *et al.*, 2018). The error bars represent Mean ± SD, the lowercase letters indicate significant difference at *P* = 0.05.

Production of volatile compounds is a major trait of ripe fruit that is highly appreciated by consumers. At the B + 10 stage, volatiles derived from different pathways (Fig. S5a) were measured by GC and GC‐MS and were significantly reduced in RIN‐deficient fruits compared to WT (Fig. S5b; Table S3). These included volatiles generated from amino acids (phenylethyl alcohol, 2‐hydroxy‐benzaldehyde, 2‐methoxy‐phenol, benzeneacetaldehyde, benzaldehyde, 2‐Isobutylthiazole), carotenoids (6‐methyl‐5‐hepten‐2‐one, (*E*)‐3‐Buten‐2‐one), lipids (hexanal, heptanal, (*E*)‐2‐heptenal, (*E*)‐2‐Pentenal) (Fig. 4a). Of the volatile compounds shown to be reduced in RIN‐deficient fruits, nine (phenylethyl alcohol, 2‐hydroxy‐benzaldehyde, benzaldehyde, benzeneacetaldehyde, 2‐isobutylthiazole, 6‐methyl‐5‐hepten‐2‐one, heptanal, (*E*)‐2‐heptenal, (*E*)‐2‐pentenal) are significantly positively correlated with consumer preferences (Zhang *et al.*, 2016; Klee and Tieman, 2018). Analysis of transcripts of key genes from the different volatile pathways showed that they were greatly reduced (Fig. 4b), including genes encoding *branched‐chain aminotransferases* (*BCAT1*) (7.9% and 9.0% of the WT level), *lipoxygenase* C (*LoxC*) (5.4%, 3.5%), *LoxB* (6.9%, 8.0%), *hydroperoxide lyase* (*HPL*) (41.4%, 48.5%), *alcohol dehydrogenase* 2 (*ADH2*) (69.5%, 72.5%), *alcohol acetyltransferase* 1 (*AAT1*) (10.4%, 6.6%) and *l‐phenylalanine ammonia lyase* (*PAL3*) (43.8%, 33.9%). Several of these genes have been identified previously as direct targets of RIN including *LoxC*, *HPL*, *AAT1* and *BCAT1*, which also could be induced by ET in RIN‐CRISPR fruits (Fujisawa *et al.*, 2012; Zhong *et al.*, 2013; Fig. 4b).

### RIN‐deficient fruits soften extensively

**Figure 5:**
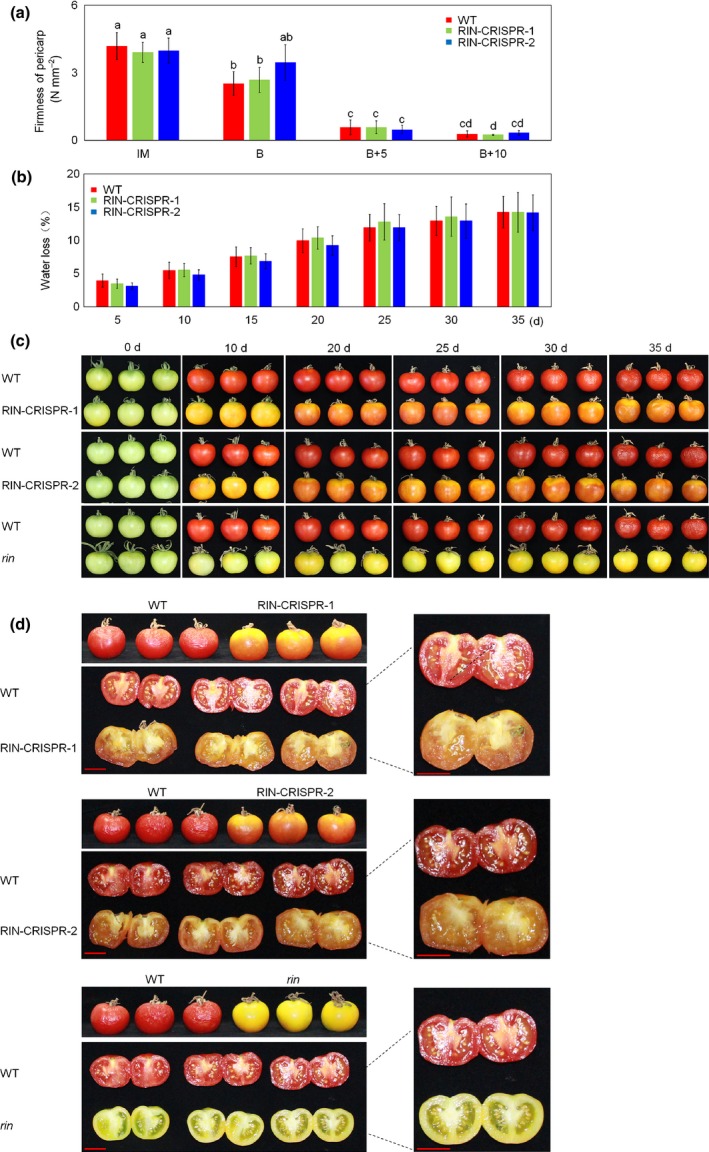
Texture change, water loss and phenotype during post‐harvest stages. (a) Fruit firmness in wild‐type (WT) and RIPENING INHIBITOR (RIN)‐CRISPR tomato measured by penetrometer. Tomato fruits from two RIN‐CRISPR homozygous mutant lines (RIN‐CRISPR‐1, RIN‐CRISPR‐2) and WT were picked at four different ripening stages, including immature (IM), breaker (B), breaker + 5 (B + 5) and breaker + 10 (B + 10). (b) Water loss by WT and RIN‐CRISPR tomato fruits during post‐harvest storage. Tomato fruits from RIN‐CRISPR‐1, RIN‐CRISPR‐2 and WT were picked at early breaker (B), and stored for 0 days (d), 10 d, 20 d, 25 d, 30 d and 35 d. Measurement of water loss was as described in Materials and Methods. The error bars represent mean ± SD. (c) Phenotype of both WT, RIN‐CRISPR and *rin* mutant tomato fruits during post‐harvest ripening and storage. Tomato fruits from RIN‐CRISPR‐1, RIN‐CRISPR‐2, *rin* mutant and WT were picked at early B stage and stored for 0 d (d), 10 d, 20 d, 25 d, 30 d and 35 d. (d) Tomato fruits from RIN‐CRISPR‐1, RIN‐CRISPR‐2, *rin* mutant and WT were picked at early B stage and stored for 35 d and photographed. Enlarged photos of representative samples are shown compared to WT fruits on the right. Bar, 2 cm.

**Figure 6:**
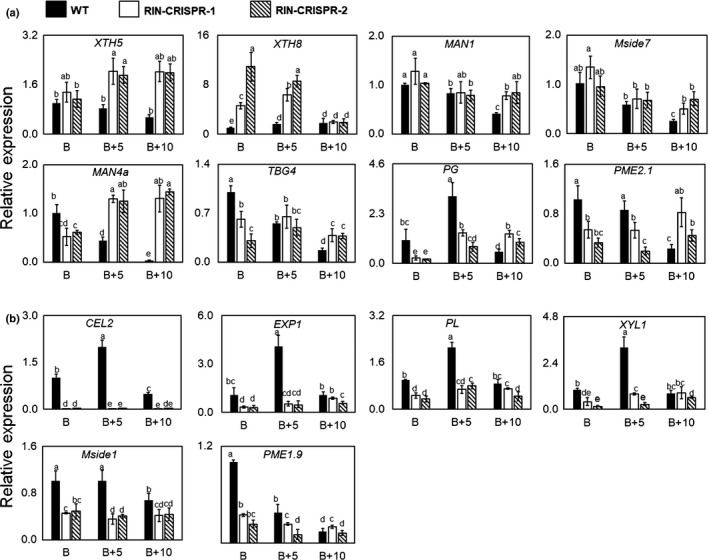
Transcripts of cell wall‐modifying enzymes in RIPENING INHIBITOR (RIN)‐deficient and wild‐type (WT) fruits measured by quantitative reverse transcription (qRT)‐PCR. (a) Genes whose transcripts are higher in RIN‐deficient fruits compared to WT fruits during at least one ripening stage. (b) Genes whose transcripts are lower in RIN‐deficient fruits compared to WT. Expression of genes was measured in fruits selected at the breaker (B), breaker + 5 (B + 5) and breaker + 10 (B + 10) stages. Transcript levels were determined by qRT‐PCR, relative to the expression of the tomato *ACTIN* gene internal control, expressed as 2^‐ΔΔCt^ (Livak & Schmittgen, 2001): *polygalacturonase* (*PG*)*, expansin 1* (*EXP1*)*, cellulase* (*CEL2*)*, pectinesterase* (*PE1/PME1.9, PME2.1*)*, pectate lyase* (*PL*)*, α‐mannosidase* (*MAN1*)*, endo‐1,4‐β‐mannanase* (MAN4a), *β‐d‐xylosidase* (*XYL1*)*, β‐mannosidase* (*Mside1*), *endo‐1,4‐β‐mannosidase 7* (*Mside7*), *tomato β‐galactosidase* (*TBG4*)*, xyloglucan endotransglucosylase/hydrolase* (*XTH5* and *XTH8*)*.* The error bars represent mean ± SD, the lowercase letters indicate significant difference at *P* = 0.05.

Softening is a major component of fleshy fruit ripening. At early ripening stages, WT and RIN‐CRISPR tomato fruits softened at a similar rate and pericarp firmness of mutant lines, measured by penetrometer, was not significantly different from that of the WT fruit from breaker (B) to breaker + 10 (B + 10) (Fig. 5a). Up to 35 d post‐harvest there was no significant difference in water loss between RIN‐deficient and WT fruits during prolonged post‐harvest storage (Fig. 5b). Transcripts of *cellulase* (*CEL2*) were greatly reduced in RIN‐deficient fruits (< 1% WT) and *polygalacturonase* (*PG*) transcripts also were initially strongly reduced but reached higher levels when fruits were picked at late stages (Fig. 6). Transcripts of other cell wall‐related genes such as *expansin 1* (*EXP1*), *pectate lyase* (*PL*), *β‐d‐xylosidase* (*XYL1*), *β‐mannosidase* (*Mside1*) and *pectinmethylesterase* (*PE1/PME1.9*) also were decreased but still accumulated to relatively high levels. *CEL2*, *EXP1, PL* and *XYL1* have all been reported to be directly targeted by RIN (Fujisawa *et al.*, 2013; Zhong *et al.*, 2013). Of particular note, transcripts of *endotransglucosylase/hydrolase* (*XTH5* and *XTH8*), which have not been identified among RIN targets (Fujisawa *et al.*, 2013; Zhong *et al.*, 2013), were significantly higher in RIN‐deficient fruits compared to WT. Over longer timescales the RIN‐deficient fruits stored less well than WT fruit (Fig. 5c). The internal structure visibly showed greater disruption and loss of integrity than WT fruits (Fig. 5d). Transcripts of other genes, such as *α‐mannosidase* (*MAN1*), *endo‐1,4‐β‐mannosidase 7* (*Mside7*), *endo‐1,4‐β‐mannanase* (*MAN4a*), *tomato β‐galactosidase* (*TBG4*), *polygalacturonase* (*PG*) and *pectinmethylesterase* (*PME2.1*) were present at higher levels at later ripening stages in RIN‐deficient fruits compared to WT (Fig. 6). Of these, *PG*, *TBG4*, *Mside7* and *MAN4a* have been reported to be directly targeted by RIN (Fujisawa *et al.*, 2013; Zhong *et al.*, 2013).

Exogenous ET treatment enhanced the expression of several cell wall‐modifying genes in WT fruit, whereas the same treatment of RIN‐deficient fruit resulted in a much smaller upregulation of some genes, including *PG*, *XYL1* and *TBG4* (Fig. S6a), whereas *Mside7* and *PME1.9* transcripts were slightly reduced by ET treatment in RIN‐deficient fruits (Fig. S6b). By contrast, transcripts of *CEL2*, *EXP1*, *PL*, *Mside1*, *MAN4a* and *PME2.1* were unaffected by supplying ET externally (Fig. S6c). Furthermore, transcripts for genes such as *XTH5*, *XTH8* and *MAN1* displayed no obvious response to ET treatment in RIN‐deficient fruits and were present at similar or higher levels than in WT (Fig. S6d) and *XTH1‐4, 6, 7* were undetectable.

## Discussion

### Ethylene is sufficient to initiate ripening in mature RIN‐deficient fruit

Ethylene (ET) has long been implicated in the control of ripening. Ethylene action inhibitors silver (Ag^+^) and later 1‐methylcyclopropene (1‐MCP) slow or inhibit ripening and there is a requirement for ET for the stable and continuous expression of ripening genes in fruit expressing RIPENING INHIBITOR (RIN) (Davies *et al.*, 1988; Grierson, 2013). Reducing expression of *ACC synthase* (*ACS*) and *ACC oxidase* (*ACO*) ET biosynthesis genes by antisense/RNAi also slowed or prevented ripening (Hamilton *et al.*, 1990; Oeller *et al.*, 1991; Picton *et al.*, 1993). Kevany *et al.* (2007) concluded that degradation of ET receptors, which are negative regulators, could influence the timing of ripening but this analysis was performed with plants expressing an intact functional *RIN* gene. A striking feature revealed by the present study is that the reduced amount of endogenous ET generated by RIN‐deficient fruits is sufficient to intitiate ripening in the absence of RIN (Figs 1, 2) as proposed by Ito *et al.* (2017). The inhibition of colour change by 1‐MCP and the responsiveness of carotenoid biosynthesis genes such as *phytoene synthase1* (*PSY1*) and others upon ET treatment (Figs 1, 3) confirms this directly and indicates that this is a genuine ET response. However, ET treatment of RIN‐deficient lines did not restore full ripening and, unlike the situation in wild‐type (WT) fruits, system‐2 ET production could not be induced by propylene in RIN‐CRISPR fruits (Fig. 2). The response to the ET analogue propylene is a classic test for initiation of autocatalytic ET synthesis during ripening (McMurchie *et al.*, 1972) and the lack of a response in RIN‐deficient tomatoes indicates that RIN is required for the induction of system‐2 ET production (Fig. 2). These results are all consistent with the suggestion that the observed RIN‐independent initiation of ripening in mature RIN‐deficient fruits is actually caused by the low endogenous ET that they produce at maturity. This does not solve the ripening initiation problem completely, however, because it is known that an increase in synthesis and responsiveness to ET only occurs in mature green (MG) fruit and does not occur in immature fruit.

### Discrepancies between fruits of the *rin* mutant and RIN‐deficient fruit

The original *rin* mutation was known to involve a deletion and fusion of parts of two adjacent genes, called *RIN* and *MC* (Vrebalov *et al.*, 2002); *rin* was thought to correspond to a loss‐of‐function mutation and the analysis of the phenotype of *rin* mutant fruits was influential in shaping the conclusions about RIN function. Recent studies, however, have shown that RIN‐MC protein is an active transcription factor (TF) with a repressor function (Ito *et al.*, 2017; Li *et al.*, 2018).

Our data show that RIN‐deficient fruits are able to initiate ripening at a similar time to WT fruit (Fig. 1), supporting the conclusion that RIN is not required for ripening initiation, although it is needed for the progression and completion of ripening (Ito *et al.*, 2017; Li *et al.*, 2018). This poses the question: what initiates ripening and what is the role of RIN in ripening progression? RIN‐deficient (RIN‐CRISPR) fruits can respond to ET treatment, as indicated by changes in transcript levels of carotenoid pathway genes, required for pigment accumulation, and the accumulation of only a very limited quantity of carotenoids, especially lycopene (Fig. 3). The involvement of ET is confirmed by the demonstration that it is inhibited by the ET perception inhibitor 1‐MCP (Fig. 1d). Our study clarifies the role of RIN in the control of climacteric ripening and highlights the importance of autocatalytic system‐2 ET production (Fig. 2) for the subsequent progression of ripening and development of quality attributes such as coloured carotenoids and volatiles. The observed differences between the original *rin* mutants and the RIN‐CRISPR lines described in the present study relate to the strong transcription repressor activity of the chimeric RIN‐MC TF generated by the mutation (Ito *et al.*, 2017; Li *et al.*, 2018). Key differences are that RIN is required for volatiles production but not softening, whereas RIN‐MC, generated by the *rin* mutation, inhibits both.

### Post‐harvest RIN‐deficient fruit soften extensively

Breeders have used the *rin* mutation in hybrids to reduce tomato fruit softening and prolong shelf life. In the present experiments, removal of RIN greatly reduced the production of ET, carotenoids and volatiles but extensive softening still occurred (Figs 5, 6). This is inconsistent with RIN being a ‘master regulator’ of all aspects of ripening. The accumulation of cell wall‐modifying enzymes is greatly reduced in *rin* mutant fruit (Knapp *et al.*, 1989; Tucker *et al.*, 2017) and this may be related to the repressor function of the RIN‐MC protein generated by the *rin* mutation (Li *et al.*, 2018). By contrast, however, in RIN‐deficient fruits we found that transcripts of some softening enzyme genes including *XTH5* and *XTH8* were expressed at higher levels compared to WT at most stages of ripening. This might explain why RIN‐deficient fruits softened in a similar way to WT fruits at early ripening stages (Fig. 5). Surprisingly, early softening occurred in the almost complete absence of *CEL2* and reduced levels of *EXP1*, *PL, XYL1, Mside1* and *PME1.9* transcripts (Fig. 6), although at later stages *MAN1*, *Mside7*, *MAN4a*, *TBG4*, *PG* and *PME2.1* transcripts were significantly more abundant than in WT (Fig. 6). The *XTH* genes, which encode xyloglucanendo‐transglucosylase/hydrolases (XTHs: EC2.4.1.207 and/or EC3.2.1.151) have been proposed to have a dual role integrating newly secreted xyloglucan chains into an existing wall‐bound xyloglucan and restructuring the existing cell wall material by catalyzing transglucosylation between previously wall‐bound xyloglucan molecules (Miedes and Lorences, 2009; Muñoz‐Bertomeu *et al.*, 2013). *XTH5* and *XTH8* are the only *XTHs* reported to be expressed in fruits (Miedes and Lorences, 2009) and the higher levels of *XTH5* and *XTH8* transcripts that we measured (Fig. 6a) might cause cell wall structural changes affecting texture or softening of RIN‐deficient fruits. The fact that these genes are expressed more highly in RIN‐deficient lines may indicate that, when present, RIN can actually repress their expression, but this needs experimental confirmation. RIN‐deficient tomato fruits showed further abnormal softening late in the ripening process (Fig. 5). This is likely to be related to the late accumulation of abnormally high levels of transcripts for several wall‐modifying enzymes, including *Mside7*, *MAN1, MAN4a, TBG4, PG* and *PME2.1* transcripts (Fig. 6). *Mside7* and *MAN1,* encoding endo‐1,4‐β‐mannosidase 7 and α‐mannosidase, respectively, are likely to affect cell wall structure, because fruits of transgenic *α‐Man* (*MAN1*) tomato RNAi lines were approx. 2.5‐fold firmer with *c.* 30 d extended shelf life, compared to WT, whereas overexpression of *α‐Man* resulted in excessive fruit softening (Meli *et al.*, 2010).

### RIN, ET acting via ET response factors (ERFs) and other TFs are involved in regulating fruit softening

Early studies showed that application of ET action inhibitors to tomatoes inhibited accumulation of *PG* and other gene transcripts (Davies *et al.*, 1988; 1990) and the present findings support the conclusion that ET enhances accumulation of *PG* transcripts (Fig. S6). This is in disagreement with results of Oeller *et al.*, (1991) but supports the conclusion of Sitrit & Bennet (1998). An ET response element (GCC‐box) is present in the *PG* gene promoter (Fig. S7). Transcripts of cell wall‐modifying genes were much lower in response to ET in RIN‐deficient fruits compared to ET‐treated WT fruits (Fig. S6). Without RIN, the fold‐increase in response to ET was similar to WT, although the actual value was much lower (Fig. S6). This may indicate that RIN and ET, acting via ERFs and possibly other TFs, are both required for maximum expression of these cell wall genes. These results support a model where RIN and ET are both required for normal softening during ripening. Direct enhancement of transcription would be expected to involve an ERF activator, but the absence of the classical ERF GCC‐box binding site in the promoters of all cell wall genes except *PG* has raised doubts about this possibility. There is, however, evidence for involvement of ERFs in recognizing alternative promoter motifs. Tomato MADS protein FUL1 has been shown to interact with ERF8 in vivo (José Ripoll *et al.*, 2015), and in Arabidopsis it has been shown that the AtRAP2.2 ERF binds an ATCTA motif and not the classical GCC‐box. We note that ATCTA motifs are present in all the promoters of the cell wall genes examined (Fig. S7). Thus, full expression of cell wall enzymes may require RIN and ERFs.

Evidence from other recent work (Gao *et al.*, 2018) has confirmed that other TFs, including NACs, also modulate expression of genes involved in cell wall metabolism. There is some evidence that RIN interacts with a NAC protein, NAC4 (Zhu *et al.*, 2014), and may also form heterodimers with other MADS TFs, such as TAGL1, AGL11 and FUL1/2 (reviewed in Li *et al.*, 2019). *Cel2*, *EXP1* and *MAN4a* gene promoters also can be direct targets of both FUL1 and FUL2, whereas *PL* and *XTH5* are targeted only by FUL1 (Fujisawa *et al.*, 2014). NOR‐like1, another NAC TF, has recently been shown to directly regulate *PG*, *PL*, *CEL1* and *EXP1* genes and also enhances ET production (Gao *et al.*, 2018). Thus, RIN, NAC(s) and an ERF that recognizes the ATCTA motif may all contribute to the regulation of the expression of tomato cell wall‐modifying genes.

### Effect of manipulation of RIN on tomato fruit volatiles

The major reduction in aroma volatiles (Figs 4, S5) in RIN‐deficient fruits was related to the reduction in transcripts of key genes that operate in three different biosynthetic pathways to generate compounds that contribute to aroma and flavour (Fig. 4b). These include *BCAT1*, which is important in the production of branched‐chain amino acids (Maloney *et al.*, 2010;Kochevenko *et al.*, 2012; Zhang *et al.*, 2016), transcripts of *LoxC*, *LoxB*, *HPL* and *ADH2* in the lipid pathway, where production of the C5 volatile 1‐penten‐3‐ol is dependent upon LoxC action (Shen *et al.*, 2014) and the C6 volatiles require sequential activity of LoxC and HPL (Chen *et al.*, 2004). *AAT1* is important in volatile esters synthesis (Yahyaoui *et al.*, 2002; Goulet *et al.*, 2015) and the final step in the pathway, conversion of aldehydes to alcohols, requires ADH2 activity (Speirs *et al.*, 1998). A major group of amino acid‐derived flavour and aroma compounds, benzenoids (C6‐C1), are synthesized from l‐phenylalanine, including benzaldehyde and benzyl alcohol (Fig. S5). The first step in the biosynthesis of these compounds is catalyzed by l‐phenylalanine ammonia lyase (PAL), which converts phenylalanine to *E*‐cinnamic acid (Aragüez and Valpuesta, 2013) and the reduction in *PAL3* transcripts in RIN‐deficient fruits is consistent with the reduced products from this pathway (Fig. 4b). *AADC1A* mediates the first step in the production of phenylalanine‐derived volatiles in tomato fruits (Tieman *et al.*, 2006). These transcripts were reduced in RIN‐deficient fruits (Fig. 4b) and this is consistent with lower concentrations of aroma volatiles from the amino acid pathway (Figs 4a, S5). Although transcripts of the *CCD1B* gene were not significantly different in RIN‐deficient fruits, the accumulation of volatiles such as (*E*)‐3‐buten‐2‐one, 6‐methyl‐5‐hepten‐2‐one were reduced (Fig. 4). These compounds are carotenoid derivatives and the severely reduced carotenoid content of RIN‐deficient fruits (Ito *et al.*, 2017; Li *et al.*, 2018) probably explains the reduced concentrations of these volatiles.

The use of the *rin* mutation by tomato breeders to generate hybrids with improved shelf‐life has an adverse effect on tomato flavour (Osorio *et al.*, 2019). Our results indicate that the normal RIN protein in WT fruit makes a major contribution to volatile formation, which suggests that if other *RIN* mutations were used for breeding, they also could have an adverse effect on flavour.

### Models for initiation and progression of ripening and generation of quality attributes

**Figure 7:**
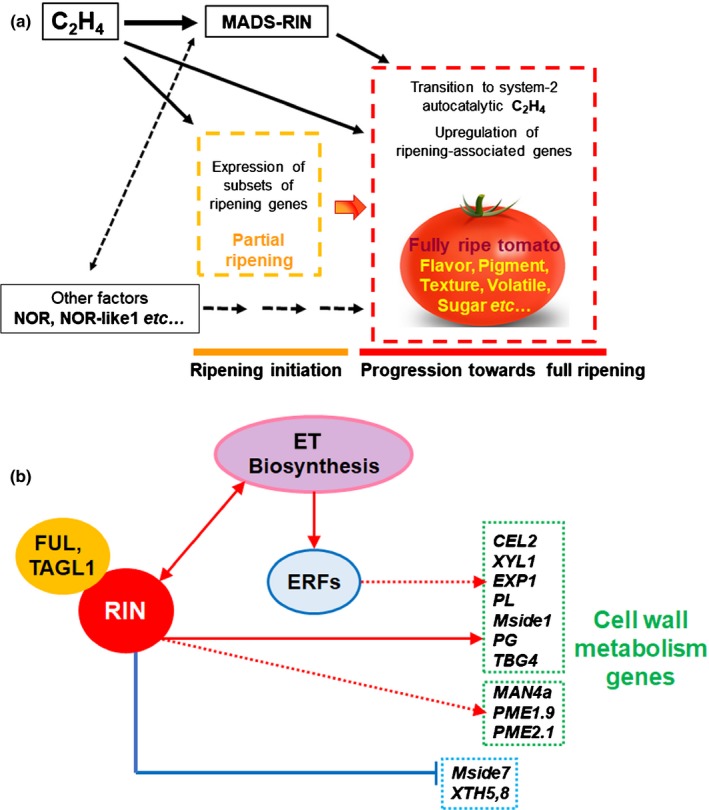
Model outlining the role of ethylene (ET) and RIPENING INHIBITOR (RIN) in initiation and progression of climacteric ripening in tomato fruit. (a) Ethylene can initiate ripening in a RIN‐independent way leading to partial ripening (data provided herein). However, RIN is required for autocatalytic system‐2 of ET production and subsequent full ripening. *RIN* expression is enhanced by ethylene (Giovannoni *et al.*, 2017; Li *et al.*, 2018; Lü *et al.*, 2018). Other factors such as NOR, NOR‐like1, ERFs, ARFs and TAGL1, not mentioned in this model, also are involved in the ripening genetic programme (Li *et al.*, 2019; Gao *et al.*, 2018). (b) A model of the role of RIN and ET in regulating tomato fruit cell wall changes and softening. Accumulation of *CEL2*, *XYL1*, *EXP1*, *PL*, *Mside1*, *PG* and *TBG4* transcripts is stimulated by ethylene (Fig. S6). RIN also regulates the transcription of genes involved in cell wall metabolism, such as *CEL2, XYL1, EXP1, PL, Mside1* and *PME1.9* (Fig. 6; S6), whereas genes such as *XTH5* and *XTH8* are expressed at a higher level in the absence of RIN. By contrast, increases in transcripts of *PG, TBG4*, *PME2.1*, *Mside7* and *MAN4a* are delayed but eventually reach very high levels in the absence of RIN (Fig. 6).

The physiological and molecular analysis of RIN‐deficient tomato fruits sheds new light on the mechanisms underlying initiation and progression of fruit ripening. First, there is a requirement for ET to initiate ripening via a RIN‐independent route (Fig. 1). Second, this leads to the induction of *RIN* (Giovannoni *et al.*, 2017; Li *et al.*, 2018; Lü *et al.*, 2018). The effect of ET on *RIN* transcripts was quantified in our previous experiments (Li *et al.*, 2018) and we found that *RIN* mRNA was reduced 4‐5‐fold by treating MG WT fruit with 1‐MCP compared to the control. RIN and TAGL1 also may be involved in a positive feedback loop leading to system‐2 ET production, because ET biosynthesis genes have been reported to be directly targeted by a RIN‐TAGL1 complex (Lü *et al.*, 2018). We propose a working model (Fig. 7a) where ET is required to initiate ripening and activate expression of a subset of ripening genes. Among these genes is the regulator MADS‐RIN, which activates a further subset of ripening genes, including those required for system‐2 autocatalytic ET synthesis, such as *ACS2*, *ACS4* and *ACO1* (Fig. S3; Liu *et al.*, 2015). However, the inability of RIN‐deficient fruits to produce system‐2 ET is insufficient to explain their failure to ripen, because these fruit do not ripen completely, even after 15 d exposure to 100 ppm ET (Fig. 1d). This suggests that other factors also may contribute to the regulation of this process, including other phytohormones such as auxin (Shin *et al.*, 2019). RIN promotes transcript accumulation from some genes involved in cell wall metabolism (Figs 6, S6) and ET stimulates the accumulation of others, discussed in the legend to Fig. 7(b). On the one hand, increases in *PG*, *TBG4*, *PME2.1*, *Mside7* and *MAN4a* transcripts are delayed but they eventually reach very high levels in RIN‐deficient fruits (Fig. 6). *XTH5* and *XTH8* transcripts, on the other hand, accumulate to higher levels in the absence of RIN, suggesting that when present RIN may cause a significant reduction in their expression. It is important to test these various possibilities in order to clarify the mechanism whereby ET stimulates expression of cell wall‐modifying and other ripening‐related genes.

## Author contributions

SL performed the experiments; DG and SL designed the experiments, wrote the article, analyzed the data, selected, designed and generated the figures; BZZ provided the transgenic and mutant tomato materials and participated in design of the study; CX and BZ assisted with the carotenoid and volatile measurements; MB contributed to the design of some experiments and to the analysis and discussion and presentation of the data; JP, MB and DG developed the model and edited the final document; KC participated in design of the study and provided support for the project and the tomato ripening program. All authors approved the article.

## Supporting information


**Fig. S1** Construction of RIN‐CRISPR tomato mutants.
**Fig. S2** Phenotype of RIN‐CRISPR tomato fruits.
**Fig. S3** qRT‐PCR assay of genes involved in ET biosynthesis, perception and signaling.
**Fig. S4** Colour development in WT and RIN‐CRISPR tomato fruits.
**Fig. S5** Biochemical origin of tomato fruit volatiles and the content in WT, RIN‐CRISPR‐1 and RIN‐CRISPR‐2 tomato fruits.
**Fig. S6** Transcripts of cell wall‐modifying enzymes in RIN‐deficient and WT fruits treated with ET and 1‐MCP measured by qRT‐PCR.
**Fig. S7** RIN and ERF binding motifs in promoters of cell wall‐metabolizing genes.
**Table S1** Primer pairs for vector construction and target site mutation analysis.
**Table S2** Primer pairs for qRT‐PCR assay.

**Table S3** Volatile components of AC, RIN‐CRISPR‐1 and RIN‐CRISPR‐2 tomato fruits at B, B+5 and B+10 stages.
Please note: Wiley Blackwell are not responsible for the content or functionality of any Supporting Information supplied by the authors. Any queries (other than missing material) should be directed to the *New Phytologist* Central Office.
