# Supplementary material for: Roles of RIN and ethylene in tomato fruit ripening and ripening‐associated traits
Source: New Phytol. 2019 Dec 31;226(2):460–75. doi: 10.1111/nph.16362 (PMC7154718; doi:10.1111/nph.16362)
Supplement: Supplementary file 1 — Fig. S1 Construction of RIN‐CRISPR tomato mutants. Fig. S2 Phenotype of RIN‐CRISPR tomato fruits. Fig. S3 qRT‐PCR assay of genes involved in ET biosynthesis, perception and signaling. Fig. S4 Colour development in WT and RIN‐CRISPR tomato fruits. Fig. S5 Biochemical origin of tomato fruit volatiles and the content in WT, RIN‐CRISPR‐1 and RIN‐CRISPR‐2 tomato fruits. Fig. S6 Transcripts of cell wall‐modifying enzymes in RIN‐deficient and WT fruits treated with ET and 1‐MCP measured by qRT‐PCR. Fig. S7 RIN and ERF binding motifs in promoters of cell wall‐metabolizing genes. Table S1 Primer pairs for vector construction and target site mutation analysis. Table S2 Primer pairs for qRT‐PCR assay. [file NPH-226-460-s001.pdf]

New Phytologist

***New Phytologist* Supporting Information**

Article title: **Roles of RIN and ethylene in tomato fruit ripening and ripening-associated traits**

Authors: Shan Li, Benzhong Zhu, Julien Pirrello, Changjie Xu, Bo Zhang, Mondher Bouzayen, Kunsong Chen, Donald Grierson

Article acceptance date: 1 December 2019

The following Supporting Information is available for this article:

Fig. S1 Construction of RIN-CRISPR tomato mutants.

Fig. S2 Phenotype of RIN-CRISPR tomato fruits.

Fig. S3 qRT-PCR assay of genes involved in ethylene biosynthesis, perception and signaling.

Fig. S4 Colour development in WT and RIN-CRISPR tomato fruits.

Fig. S5 Biochemical origin of tomato fruit volatiles and the content in WT, RIN-CRISPR-1 and RIN-CRISPR-2 tomato fruits.

Fig. S6 Transcripts of cell wall modifying enzymes in RIN-deficient and WT fruits treated with ethylene and 1-MCP measured by qRT-PCR.

Fig. S7 RIN and ERF binding motifs in promoters of cell wall metabolising genes.

Table S1 Primer pairs for vector construction and target site mutation analysis.

Table S2 Primer pairs for qRT-PCR assay.

Fig. S1. Construction of RIN-CRISPR tomato mutants. (a) Target sites designed for CRISPR/Cas9-mediated mutagenesis in the *RIN* gene. Boxes indicate exons and broken lines indicate introns. Three 20 bp target sequences and the protospacer-adjacent motif (PAM) are displayed in red color. (b) DNA editing and translation analysis in homozygous mutation alleles. DNA sequences in red color indicate the difference in *RIN* loci between WT and RIN-CRISPR mutant. The asterisks after amino acids indicate the predicted translation stop site.

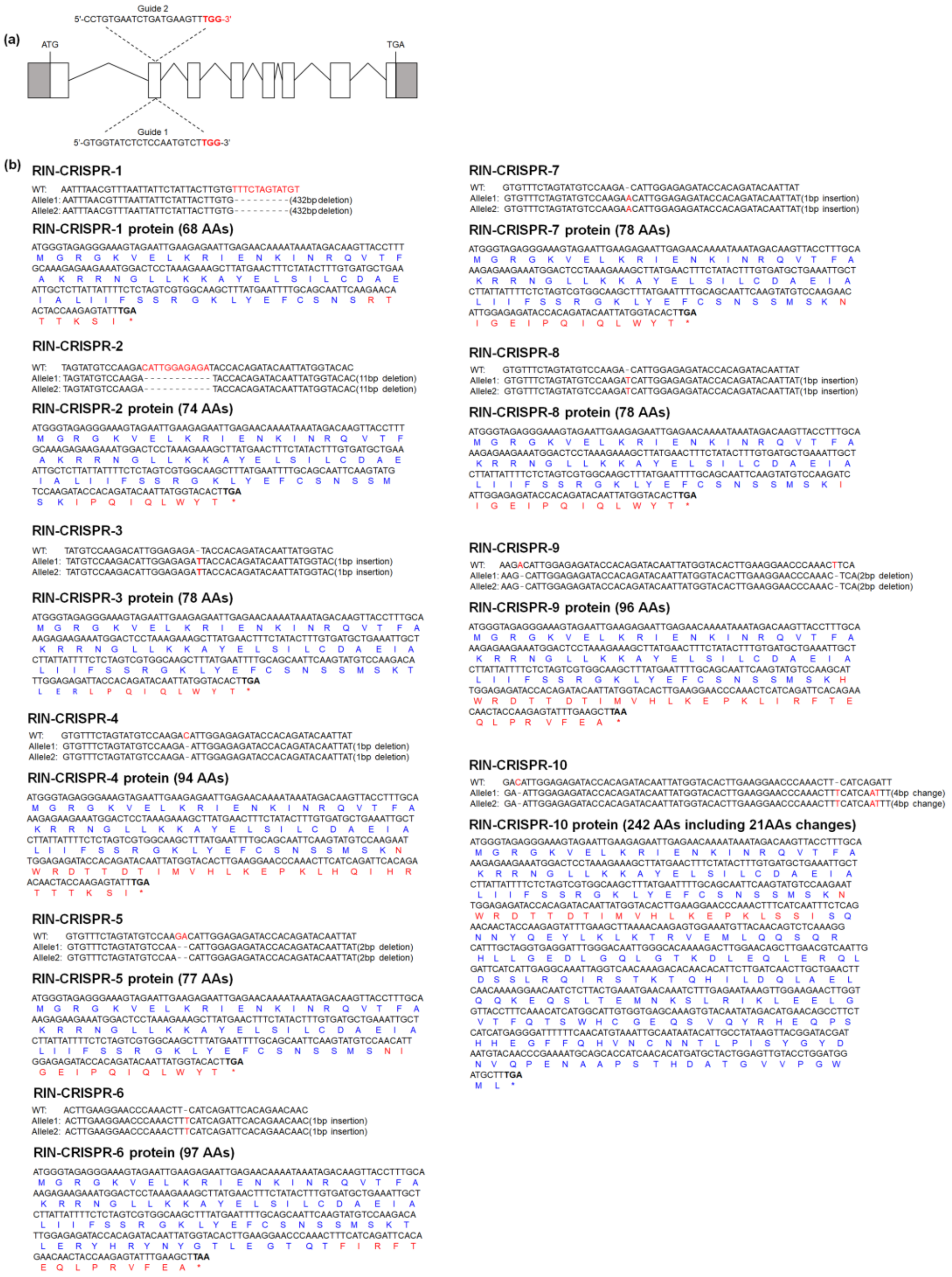

Fig. S2. Phenotype of RIN-CRISPR tomato fruits.

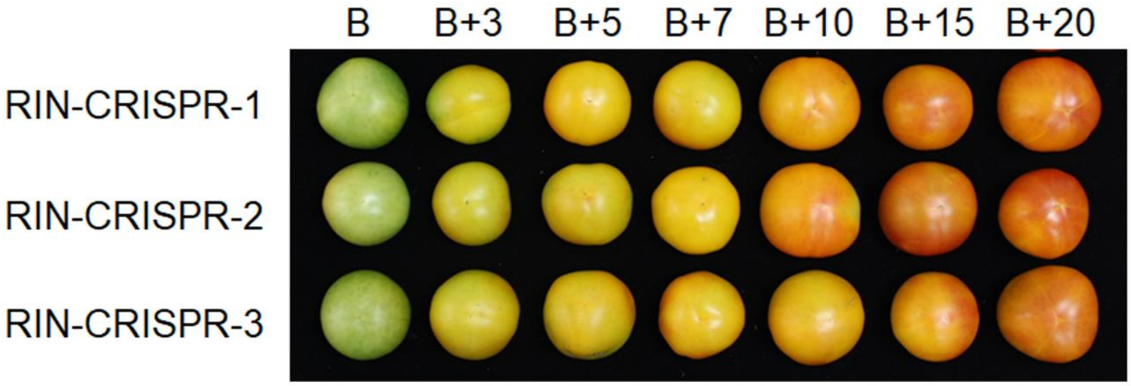

Fig. S3 qRT-PCR assay of genes involved in ethylene biosynthesis, perception and signaling. Analysis of qRT-PCR expression assay of genes involved in ethylene biosynthesis and perception (average of 3 biological replicates). Relative transcript levels were determined by qRT-PCR, relative to the expression of the tomato *ACTIN* gene internal control, expressed as $2^{-\Delta\Delta Ct}$ (Livak and Schmittgen, 2001). ACC synthesis (*ACS2*, *ACS4*); ACC oxidase (*ACO1*); Ethylene receptor (*ETR3/NR*, *ETR4*); Ethylene-response related (*E4*, *E8*); EIN3-like proteins (*EILs*); Ethylene response factors (*ERFs*). The error bars represent Mean +/- SD, the lowercase letters indicate significant difference at P=0.05.

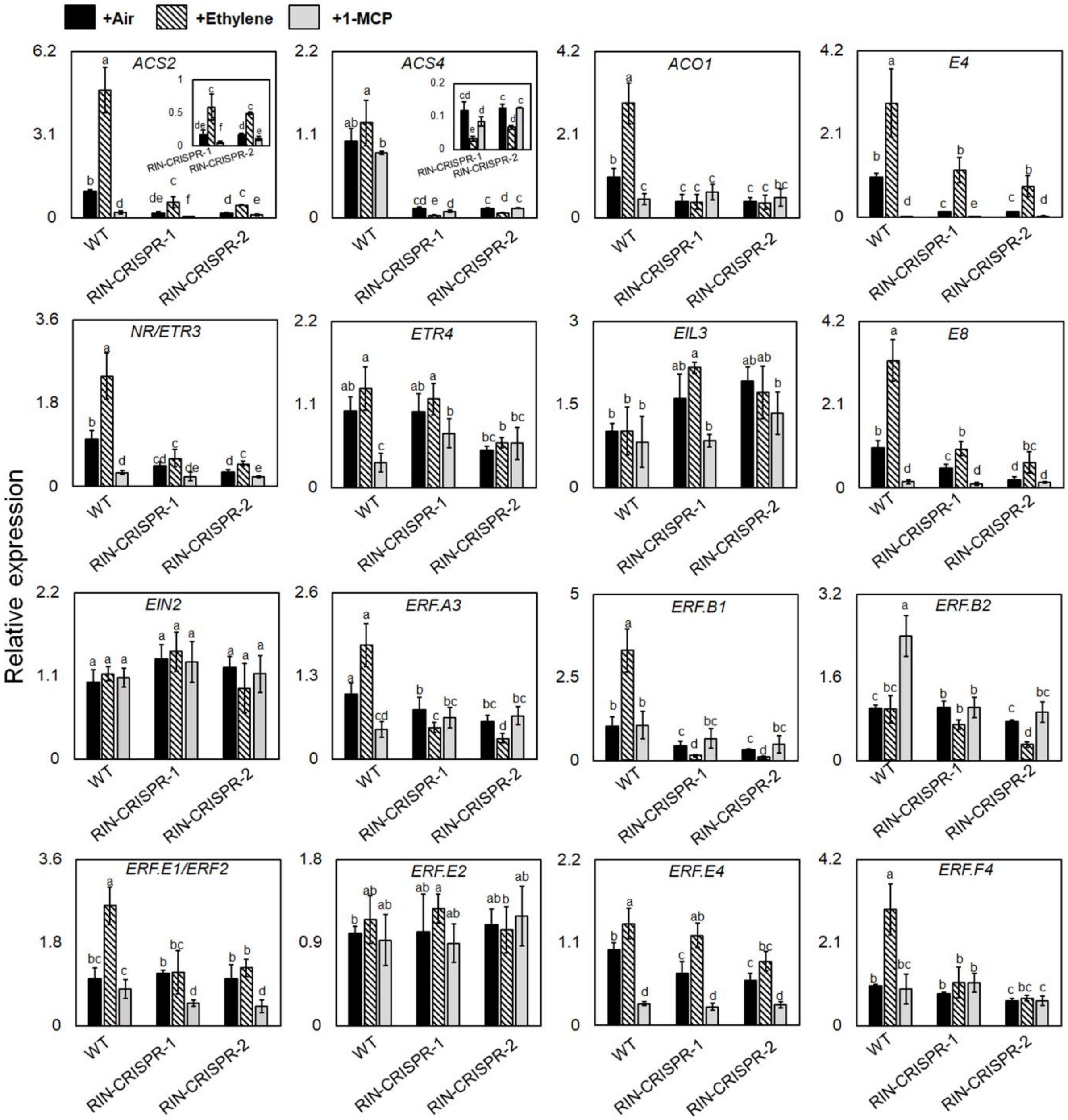

Fig. S4. Colour development in WT and RIN-CRISPR tomato fruits. Pericarp colour (a*, L*, b*), measured by handheld colorimeter using the CIE L*a*b colour system, a* indicates red to green, L* indicates lightness, b* indicates yellow to blue (Komatsu *et al.*, 2016). The error bars represent Mean +/- SD, the lowercase letters indicate significant difference.

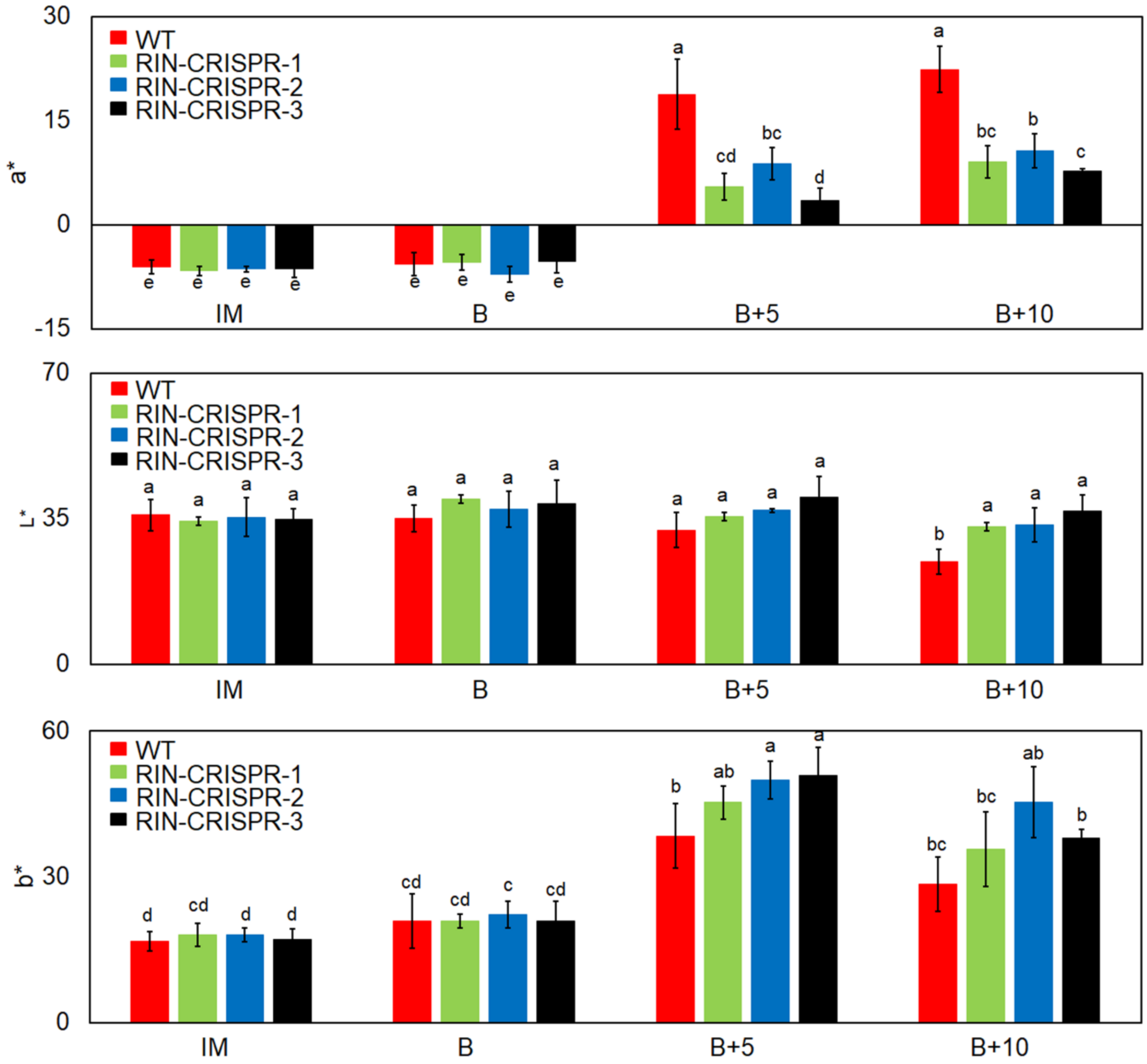

Fig. S5. Biochemical origin of tomato fruit volatiles and the content in WT, RIN-CRISPR-1 and RIN-CRISPR-2 tomato fruits. (a) The information of major pathways is from Klee and Tieman (2018) and Aragüez and Valpuesta (2013). Compounds known to be important flavor volatiles are highlighted in red, and enzymes are shown in grey boxes. (b) Table of volatile content derived from different precursors, lipid, amino acid and carotenoid.

(a)

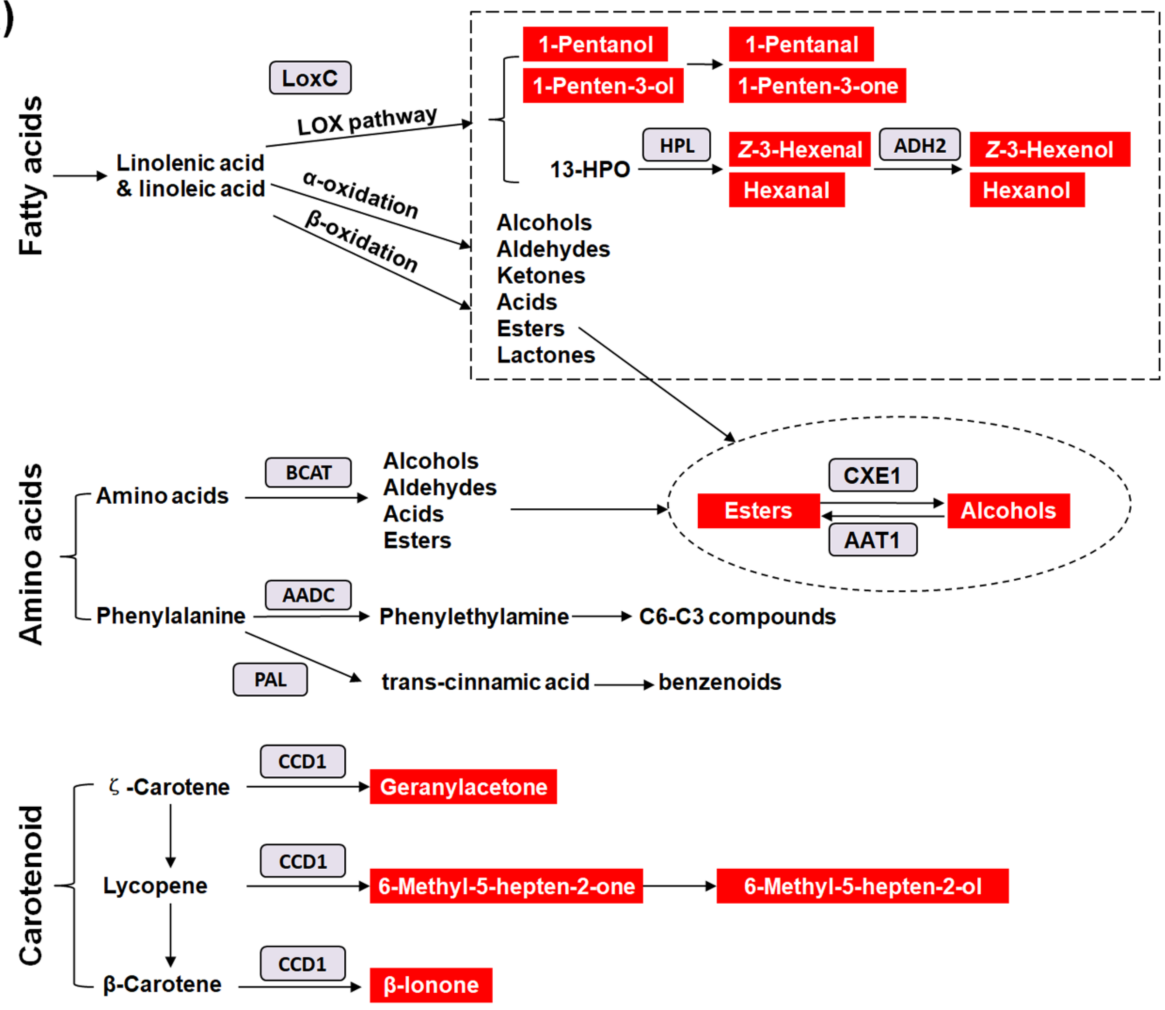

(b)

Content (μg·g-1FW)	WT			RIN-CRISPR-1						RIN-CRISPR-2					
	B	B+5	B+10	B	Change	B+5	Change	B+10	Change	B	Change	B+5	Change	B+10	Change
Lipid derivatives	11.00^{cde}	27.45^{ab}	38.18^{a}	8.74^{de}	--	16.19^{bcd}	--	16.40^{bc}	-57%	8.41^{e}	--	18.09^{bc}	--	27.58^{ab}	--
Amino acid derivatives	0.51^{d}	1.20^{b}	1.79^{a}	0.71^{cd}	--	0.84^{c}	-30%	0.74^{cd}	-59%	0.85^{bc}	20%	0.81^{c}	-33%	1.06^{bc}	-41%
Carotenoid derivatives	1.70^{c}	15.12^{a}	4.23^{b}	0.96^{d}	-43%	0.89^{de}	-94%	2.41^{c}	-43%	0.31^{f}	-82%	0.64^{e}	-96%	2.27^{c}	-46%

The lowercase letters indicate significant difference between six groups in each row.

Fig. S6 Transcripts of cell wall modifying enzymes in RIN-deficient and WT fruits treated with ethylene and 1-MCP measured by qRT-PCR. Tomato fruits were picked at mature green (MG) stage, either treated with air, ethylene or 1-MCP for 48h as described in Materials and Methods. (a) Genes whose transcripts are increased to some extent by ethylene in both RIN-deficient and WT fruits. (b) Genes whose transcripts are repressed to some extent by ethylene in RIN-deficient fruits. (c) Genes whose transcripts displayed no obvious response to ethylene, but accumulated to a lower level than WT. (d) Genes whose transcripts displayed no obvious response to ethylene, but were present at an equivalent or higher level in RIN-deficient fruits compared to WT. Transcript levels were determined by qRT-PCR, relative to the expression of the tomato *ACTIN* gene internal control, expressed as $2^{-\Delta\Delta Ct}$ (Livak and Schmittgen, 2001). The error bars represent Mean +/- SD, the lowercase letters indicate significant difference at P=0.05.

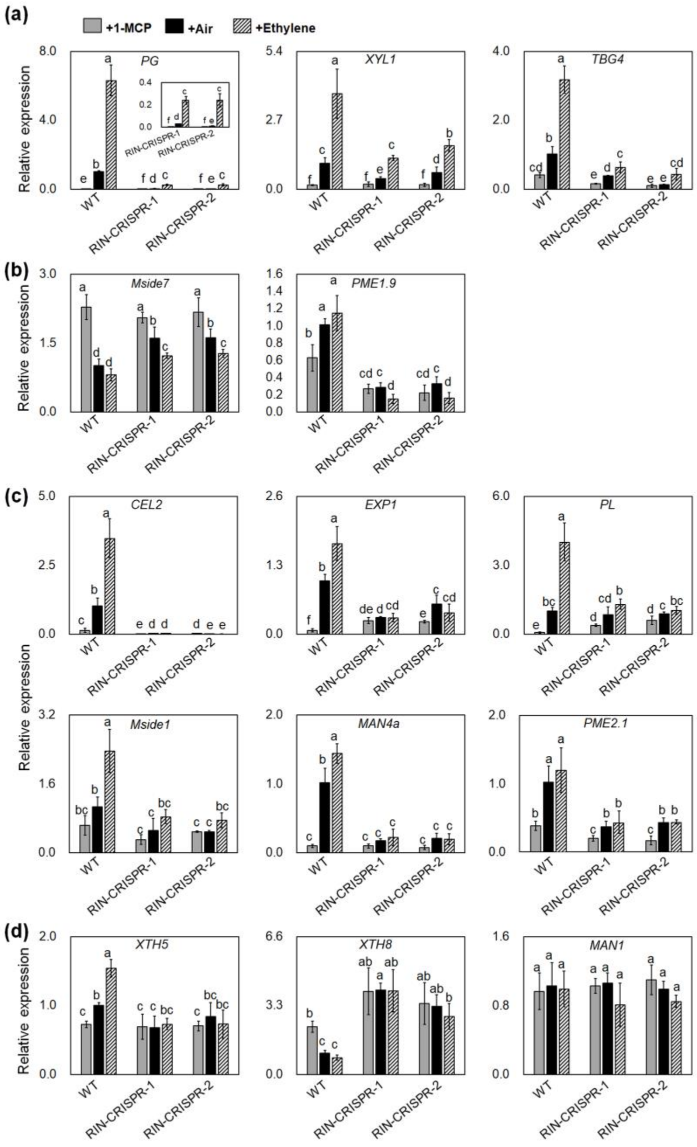

Fig. S7 RIN and ERF binding motifs in promoters of cell wall metabolising genes. Multiple CArG-box elements (MADS-box binding motifs, various CArG-box types (Fujisawa *et al.*, 2013) are present in the promoter regions (2.0 kb upstream of the ATG site) of cell wall genes, with the exception of *PL*, for which only 1.5kb promoter is available. The *PG* promoter has one GCC-box (GCCGCC, a putative AP2/ERF binding motif). A second AP2/ERF binding motif (ATCTA) described by Welsch *et al.* (2007) is also present in all cell wall genes examined in this study.

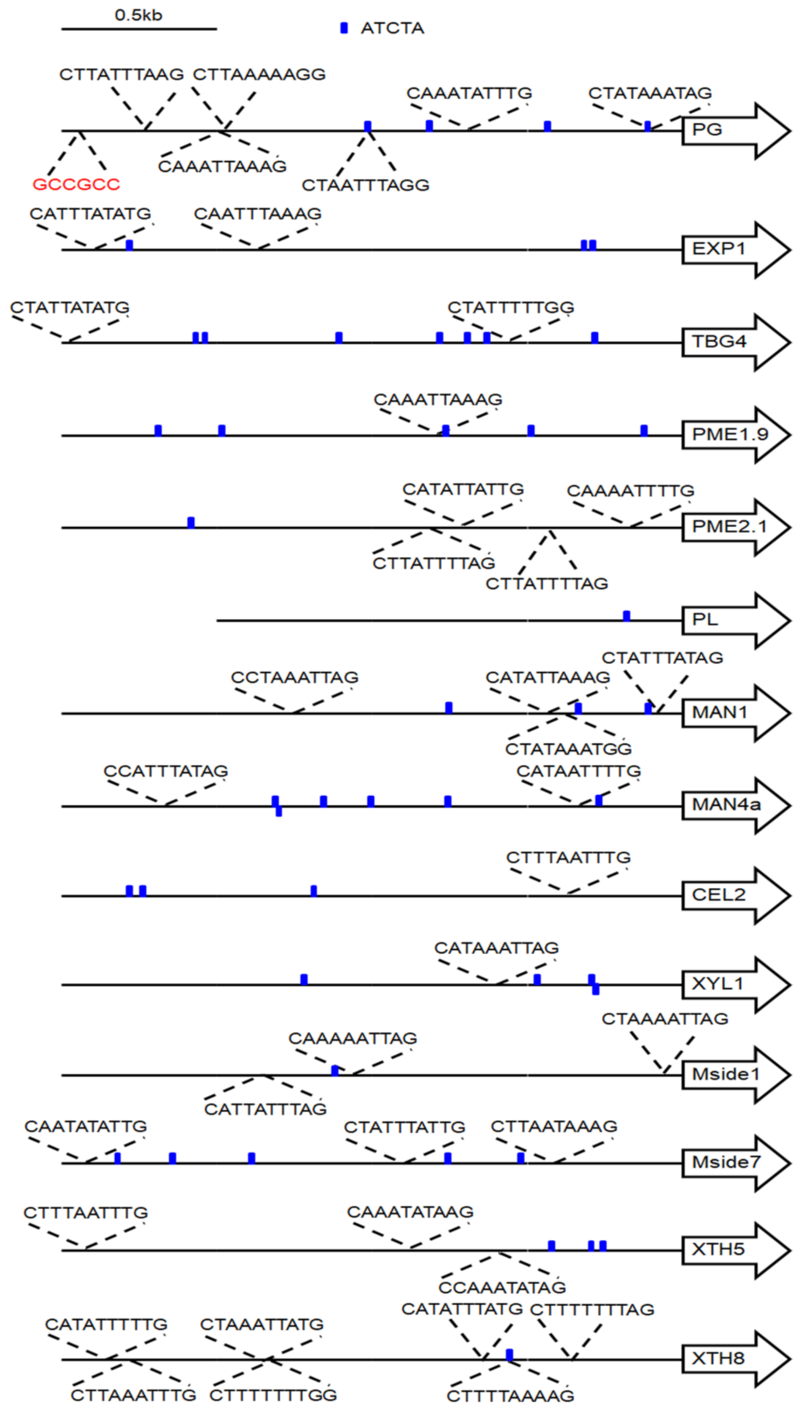

Table S1 Primer pairs for vector construction and target site mutation analysis.

Name	Primer	Sequence (5'-3’)
1st PCR	U-F	CTCCGTTTTACCTGTGGAATCG
	gR-R	CGGAGGAAAATTCCATCCAC
	gRT-RIN-T1 For	GTGGTATCTCTCCAATGTCT GTTTTAGAGCTAGAAAT
	AtU3d-RIN-T1 Rev	AGACATTGGAGAGATACCAC TGACCAATGGTGCTTTG
	gRT-RIN-T2 For	CCTGTGAATCTGATGAAGTT GTTTTAGAGCTAGAAAT
	AtU3bT-T2 Rev	AACTTCATCAGATTCACAGG TGACCAATGTTGCTCC
2nd PCR	Pps-GGL	TTCAGAGGTCTCTCTCGACTAGTATGGAATCGGCAGCAAAGG
	Pgs-GG2	AGCGTGGGTCTCGTCAGGGTCCATCCACTCCAAGCTC
	Pps-GG2	TTCAGAGGTCTCTCTGACACTGGAATCGGCAGCAAAGG
	Pgs-GGR	AGCGTGGGTCTCGACCGACGCGTATCCATCCACTCCAAGCTC
Target site mutation analysis	Cas9-RIN-For	TATCAGTTGAGCACTTCGTTTT
	Cas9-RIN-Rev	GTCGTAATTTCGTGAGTTTCAT
	Cas9-RIN-Seq	GAGTTGGAATACTTAGTTGTC

New Phytologist

Table S2 Primer pairs for qRT-PCR assay.

ID	Gene	Name	Sequence(5'-3')	Reference
Solyc03g078400	*ACTIN*	ACTIN-F	CCTCAGCACATTCCAGCAG	Powell *et al.*, 2012
		ACTIN-R	CCACCAAACTTCTCCATCCC	
Solyc01g095080	*ACS2*	ACS2-F	TGGATGATGGAACGGTTGATATTGC	Li *et al.*, 2018
		ACS2-R	CCATTGTTGCTTCTGTTCCATCGAAC	
Solyc05g050010	*ACS4*	ACS4-F	AAATCTCCACCTTCACTAACGAAC	Martel *et al.*, 2011
		ACS4-R	CCTAAGTCCTTGGAAAGACTAGACAC	
Solyc07g049530	*ACO1*	ACO1-F	CTTGAGGTGATCACTAACGGGAAGTACA	Li *et al.*, 2018
		ACO1-R	TGCTGGATATATTACTGCATCACTTCCTG	
Solyc09g075440	*NR*	ETR3/NR-F	ATCAGGTTGCTGTCGCTCTT	Li *et al.*, 2018
		ETR3/NR-R	GGCCATCTCTGCTTCTTGTC	
Solyc06g053710	*ETR4*	ETR4-F	AGCAATAATCATTCTCAGCCAG	Li *et al.*, 2018
		ETR4-R	TCTCCTTGCATCAACTGAACC	
Solyc03g111720	*E4*	E4-F	GACCACTCTAAATCGCCAGG	Liu *et al.*, 2018
		E4-R	TTCCTGAGCGGTATTGCTTT	
Solyc09g089580	*E8*	E8-F	TGGCTCCGAATCCTCCCAGTCT	Liu *et al.*, 2018
		E8-R	GTCCGCCTCTGCCACTGAGC	
Solyc09g007870	*EIN2*	EIN2-F	CTCAACCGTCTTCAGGGCATAG	Wang *et al.*, 2016
		EIN2-R	GTGGCGGCAAGTTTCCATTAG	
Solyc01g096810	*EIL3*	EIL3-F	CACGAGCCTCTCTTCTTCTTAC	Wang *et al.*, 2016
		EIL3-R	ACCGGCTTATGCTCAACTTC	
Solyc03g123760	*PDS*	PDS-F	CATTGATTATCCAAGACCAGAG	Wang *et al.*, 2016
		PDS-R	CCAGCAATAACAATCTCCAA	
Solyc03g031860	*PSY1*	PSY1-F	ATGTCTGTTGCCTTGTTATG	Li *et al.*, 2018
		PSY1-R	TTCCACCACCTCTATTGATT	
Solyc01g097810	*ZDS*	ZDS-F	TCTTGCTGGCTCATATACA	Li *et al.*, 2018
		ZDS-R	AGACTCAACTCATCAGATAGG	
Solyc10g081650	*CRTISO*	CRTISO-F	ATGAAGCGAAGAAAGAGGTTGT	Li *et al.*, 2018
		CRTISO-R	GCAAGGTATCGTCTGTGGGTCT	
Solyc04g079960	*GGPS2*	GGPPS2-F	TGAGGTTGATGTGGAGCGAATG	Li *et al.*, 2018
		GGPPS2-R	TAGGTCCTTACCCGCTGTCTTT	
Solyc03g007960	*SlBCH2/CHY*	CHY/SlBCH2-F	TCCGTCATAACCCGTTTCTCAG	Li *et al.*, 2018
		CHY/SlBCH2-R	CGCCAATCTCGGCTTTCTTCT	
Solyc01g067890	*DXS1*	SlDXS-F	AGCTTCCGGCTGGAAACAAA	Galpaz *et al.*, 2008
		SlDXS-R	CTAGCACAATAGCAGCATCC	
Solyc02g090890	*ZEP*	ZEP-F	CGGGTCCATCTCACATACAAAT	Moon *et al.*, 2011 (revised)

		ZEP-R	CCATGTTCACTTCGTAAATCAG	
Solyc05g052050	*ERF.A3*	ERF.A3-F	GCGAAATGGATCAACAGTTACCA	Liu *et al.*, 2014
		ERF.A3-R	ATTAGACGACTGAAGCTTGAATTCC	
Solyc05g052040	*ERF.B1*	ERF.B1-F	GAATGATGACGGAATTGTAATGAAGA	Liu *et al.*, 2014
		ERF.B1-R	TTCCACAATCCCAAATTGAAGA	
Solyc03g093560	*ERF.B2*	ERF.B2-F	AGTTTGCAGCGGAGATTCGT	Liu *et al.*, 2014
		ERF.B2-R	TGCCCTGTCATATGCCTTTG	
Solyc09g075420	*ERF.E1/ERF2*	ERF.E1-F	GTTCCTCTCAACCCCAAACG	Liu *et al.*, 2014, 2016
		ERF.E1-R	TTCATCTGCTCACCACCTGTAGA	
Solyc06g063070	*ERF.E2*	ERF.E2-F	ACTTCGTGAGGAAACCCTGAAC	Liu *et al.*, 2014
		ERF.E2-R	GTTACTAATATAAGTCATGTTGGGCTGAA	
Solyc01g065980	*ERF.E4*	ERF.E4-F	AGGCCAAGGAAGAACAAGTACAGA	Liu *et al.*, 2014
		ERF.E4-R	CCAAGCCAAACGCGTACAC	
Solyc07g053740	*ERF.F4*	ERF.F4-F	GAGCTAATGGCTGATTTTTGTATATAAGTTC	Liu *et al.*, 2014
		ERF.F4-R	AAATGGTAGAAACAGCACGAGAAAG	
Solyc06g059740	*ADH2*	ADH2-F	ATGTGTCCATGATGGCTGGG	Wang *et al.*, 2016
		ADH2-R	GGTGATGATGCAACGAAGGC	
Solyc08g014000	*LoxA*	LoxA-F	GAGGCGTGGGATAGGA	Hu *et al.*, 2014
		LoxA-R	GGATACGGGTAGTCAGCA	
Solyc01g099190	*LoxB*	LoxB-F	TGCTACAATGACTTGGGTGAA	Zhu *et al.*, 2014
		LoxB-R	CCTGTCCTGCCTCTACG	
Solyc01g006540	*LoxC*	LoxC-F	GTGCAAATACCATTAAGGCTGTG	Li *et al.*, 2018
		LoxC-R	AGTCCAGTCTTATGATCAAGCTC	
Solyc01g087260	*CCD1B*	LeCCD1B-F	AGTTCTTCCACGCTACGCAAA	Simkin *et al.*, 2004 (revised)
		LeCCD1B-R	TCTCCCTCCTCCCAAGCAT	
Solyc07g049690	*HPL*	HPL-F	AGTGAGAGACAAAGTCGGCG	Wu *et al.*, 2018
		HPL-R	ACCACAAAGAAGCTCCCCTT	
Solyc08g005770	*AAT1*	AAT1-F	CAATAAATTACCACAAGCCAAAAC	Goulet *et al.*, 2015
		AAT1-R	AACATTAGTATGGGGATTTGGAG	
Solyc12g088220	*BCAT1*	SlBCAT1-F	GTGTTGCTCCTGTAGGGAGT	Wu *et al.*, 2018
		SlBCAT1-R	AATCCAACCCCTCTTGTCCTC	
Solyc08g068680	*AADC1A*	AADCA1-F	AGCGCGACGACGATTGTT	Tieman *et al.*, 2006
		AADCA1-R	GGTCCTGCACCTGGTTGTG	
Solyc09g007900	*PAL3*	PAL3-F	CAATACTCTGCTTCAAGGCTAC	designed
		PAL3-R	CCAGCAATGTATGACAACGGG	
Solyc10g080210	*PG2a*	PG2a-F	TCAGCAAAGAGCCCAAATACTG	Li *et al.*, 2018
		PG2a-R	GCTTCCAATACTTATACCATGACCT	

Solyc06g051800	*EXP1*	EXP1-F	TGGTTCCTTCTCATTGGCAATTTGG	Martel *et al.*, 2011
		EXP1-R	TTCAGTGAGGACTCGATTTCTTTTCC	
Solyc07g064170	*PE1/PME 1.9*	PME1.9/PE1-F	GCTTGCGTCTTTGACAACTCAGG	Zhang *et al.*, 2018
		PME1.9/PE1-R	GTGCCACCACTGCATTCGCTAT	
Solyc07g064180	*PME2.1*	PME2.1-F	TCCATTACAGCACAAGGAAGACA	Jeong *et al.*, 2018
		PME2.1-R	TGTTGCAACTCTTGTTGGCT	
Solyc10g047030	*XYL1*	XYL1-F	TGATCGGCAATTATGAAGGTATTC	Zhang *et al.*, 2018
		XYL1-R	CAGCACATCCTGGCTTGTAAAT	
Solyc01g081060	*XTH5*	XTH5-F	CCACCACCAGAGTGCGAGAT	Zhang *et al.*, 2018
		XTH5-R	TTTTCTTAGGATGACGATGTCCG	
Solyc04g008210	*XTH8*	XTH8-F	TCCCAAGTGTGATATAGTCCTGGATTCTG	Muñoz-Bertomeu *et al.*, 2013
		XTH8-R	CCTCGGAGGCGATTAGCTTCCTTA	
Solyc09g010210	*CEL2*	CEL2-F	ACACATTGCCAAACGTCAGGT	Zhang *et al.*, 2018
		CEL2-R	CCCCTATGGTGAATCCTTTGTG	
Solyc03g111690	*PL*	PL-F	GCGATCAGGAGTTAGAACTGG	Zhang *et al.*, 2018
		PL-R	AATCCCCTTTTGCTTTGGTT	
Solyc12g008840	*TBG4*	TBG4-F	AAATGGTGAAGGCGTAGGTCG	Zhang *et al.*, 2018
		TBG4-R	AGGTTGTCCGCAGTTAGTCTGG	
Solyc06g068860	*MAN1*	MAN1-F	ACACCGTCCTCCTGAGATTGG	Zhang *et al.*, 2018
		MAN1-R	GAGCCTCTGCTTTCCACTTTAATC	
Solyc01g008710	*MAN4a*	MAN4a-F	TGGAGATTGGACTTGAAGGAT	designed
		MAN4a-R	CTTGAACTTGATTGTTGGAGAT	
Solyc03g119080	*Mside1*	Mside1-F	TGGAGATTGGACTTGAAGGAT	designed
		Mside1-R	CTTGAACTTGATTGTTGGAGAT	
Solyc02g084990	*Mside7*	MAN7-F	GAACTGATTTCATCGCCAAC	designed
		MAN7-R	TTGAGCATCTTGAATGTGGG	

Reference

Aragüez I, Valpuesta V (2013) Metabolic engineering of aroma components in fruits. *Biotechnol J* **8**: 1144-1158.

Fujisawa M, Nakano T, Shima Y, Ito Y (2013) A large-scale identification of direct targets of the tomato MADS-Box transcription factor RIPENING INHIBITOR reveals the regulation of fruit ripening. *Plant Cell* **25**: 371-386.

Galpaz N, Wang Q, Menda N, Zamir D, Hirschberg J (2008) Abscisic acid deficiency in the tomato mutant high-pigment 3 leading to increased plastid number and higher fruit lycopene content. *Plant J* **53**: 717-730.

Goulet C, Kamiyoshihara Y, Lam N, Richard T, Taylor M, Tieman D, Klee H (2015) Divergence in the enzymatic activities of a tomato and Solanum pennellii alcohol acyltransferase impacts fruit volatile ester composition. *Mol Plant* **8**: 153-162.

Hu T, Zeng H, Hu Z, Qv X, Chen G (2014) Simultaneous silencing of five lipoxygenase genes increases the contents of α-linolenic and linoleic acids in tomato. Solanum lycopersicum L. fruits. *J Agric Food Chem* **62**:11988-11993.

Jeong HY, Nguyen HP, Eom SH, Lee C (2018) Integrative analysis of pectin methylesterase (PME) and PME inhibitors in tomato (*Solanum lycopersicum*): Identification, tissue-specific expression, and biochemical characterization. *Plant Physiol Biochem* **132**: 557-565.

Klee HJ, Tieman DM (2018) The genetics of fruit flavour preferences. *Nat Rev Genet* **19**: 347-356.

Komatsu T, Mohammadi S, Busa LS, Maeki M, Ishida A, Tani H, Tokeshi M (2016) Image analysis for a microfluidic paper-based analytical device using the CIE L*a*b* color system. *Analyst Lond* **141**: 6507-6509

Li S, Xu H, Ju Z, Cao D, Zhu H, Fu D, Grierson D, Qin G, Luo Y, Zhu B (2018) The RIN-MC fusion of MADS-Box transcription factors has transcriptional activity and modulates expression of many ripening genes. *Plant Physiol* **176**: 891-909.

Liu MC, Chen Y, Chen Y, Shin JH, Mila I, Audran C, Zouine M, Pirrello J, Bouzayen M (2018) The tomato ethylene response factor Sl-ERF.B3 integrates ethylene and auxin signalling via direct regulation of Sl-Aux/IAA27. *New Phytol* **219**: 631-640.

Liu MC, Diretto G, Pirrello J, Roustan JP, Li ZG, Giuliano G, Regad F, Bouzayen M (2014) The chimeric repressor version of an ethylene response factor ERF family member, Sl-ERF.B3, shows contrasting effects on tomato fruit ripening. *New Phytol* **203**: 206-218.

Liu MC, Gomes BL, Mila I, Purgatto E, Peres L, Frasse P, Maza E, Zouine M, Roustan JP, Bouzayen M, Pirrello J (2016) Comprehensive profiling of ethylene response factor expression identifies ripening-associated ERF genes and their link to key regulators of fruit ripening in tomato. *Plant Physiol* 170: 1732-1744.

Livak KJ, Schmittgen TD (2001) Analysis of relative gene expression data using real-time quantitative PCR and the 2(-Delta Delta C(T)) method. *Methods* **25**: 402-408.

Martel C, Vrebalov J, Tafelmeyer P, Giovannoni J (2011) The tomato MADS-Box transcription factor RIPENING INHIBITOR interacts with promoters involved in numerous ripening processes in a COLORLESS NONRIPENING-dependent manner. *Plant Physiol* **157**: 1568-1579.

Moon Y, Lee M, Tovuu A, Lee C, Chung B, Park Y, Kim J (2011) Acute exposure to UV-B sensitizes cucumber, tomato, and Arabidopsis plants to photooxidative stress by inhibiting thermal energy dissipation and antioxidant defense. *J Radiat Res* **52**: 238-248.

Muñoz-Bertomeu J, Miedes E, Lorences EP (2013) Expression of xyloglucan endotransglucosylase/hydrolase (XTH) genes and XET activity in ethylene treated apple and tomato fruits. *J Plant Physiol* **170**: 1194-1201.

Powell A, Nguyen C, Hill T, Cheng K, Figueroa-Balderas R, Aktas H, Ashrafi H, Pons C, Fernandez-Munoz R, Vicente A, Lopez-Baltazar J, Barry C, Liu Y, Chetelat R, Granell A, Van Deynze A, Giovannoni J, Bennett A (2012) Uniform ripening encodes a Golden 2-like transcription factor regulating tomato fruit chloroplast development. *Science* **336**: 1711-1715.

Simkin A, Schwartz S, Auldridge M, Taylor M, Klee H (2004) The tomato carotenoid cleavage dioxygenase 1 genes contribute to the formation of the flavor volatiles beta-ionone, pseudoionone, and geranylacetone. *Plant J* **40**: 882-892.

Tieman D, Taylor M, Schauer N, Fernie AR, Hanson AD, Klee HJ (2006) Tomato aromatic amino acid decarboxylases participate in synthesis of the flavor volatiles 2-phenylethanol and 2-phenylacetaldehyde. *PNAS* **103**: 8287-8292.

Wang RH, Yuan XY, Meng LH, Zhu BZ, Zhu HL, Luo YB, Fu DQ (2016) Transcriptome analysis provides a preliminary regulation route of the ethylene signal transduction component, SlEIN2, during tomato ripening. *Plos One* **11**: e0168287.

Welsch R, Maass D, Voegel T, DellaPenna D, Beyer P (2007) Transcription factor RAP2.2 and its interacting partner SINAT2: stable elements in the carotenogenesis of Arabidopsis leaves. *Plant Physiol* **145**: 1073-1085.

Wu Q, Tao X, Ai X, Luo Z, Mao L, Ying T, Li L (2018) Contribution of abscisic acid to aromatic volatiles in cherry tomato *Solanum lycopersicum L*. fruit during postharvest ripening. *Plant Physiol Biochem* **130**: 205-214.

Zhang L, Zhu M, Ren L, Li A, Chen G, Hu Z (2018) The SlFSR gene controls fruit shelf-life in tomato. *J Exp Bot* **12**: 2897-2909.

Zhu M, Chen G, Zhou S, Tu Y, Wang Y, Dong T, Hu Z (2014) A new tomato NAC (NAM/ATAF1/2/CUC2) transcription factor, SlNAC4, functions as a positive regulator of fruit ripening and carotenoid accumulation. *Plant Cell Physiol* **55**:119-135.
